# mTORC1/ERK1/2 Interplay Regulates Protein Synthesis and Survival in Acute Myeloid Leukemia Cell Lines

**DOI:** 10.3390/biology12050676

**Published:** 2023-05-02

**Authors:** Concetta Anna Germano, Giuseppe Clemente, Antonello Storniolo, Maria Anele Romeo, Elisabetta Ferretti, Mara Cirone, Livia Di Renzo

**Affiliations:** Department of Experimental Medicine, Sapienza University of Rome, Viale Regina Elena 324, 00161 Rome, Italymariaanele.romeo@uniroma1.it (M.A.R.);

**Keywords:** AML, ERK1/2, mTORC1, AKT, quercetin, rapamycin, P70S6K, 4EBP1, autophagy, eIF2α

## Abstract

**Simple Summary:**

Tumor cell survival depends on the inter-connected activation of multiple pathways. Here, we show that acute myeloid leukemia cell lines benefit from an important inter-play between mTORC1-ERK and AKT, explaining why mTOR inhibition is rather ineffective in killing those cells. In fact, ERK1/2 activation, caused by the inhibition of the mTORC1-P70S6K dependent inhibitory loop, did not allow an efficient killing of AML cell lines. However, even ERK1/2 inhibition, together with that of mTORC1, caused a partial dephosphorylation of 4EBP1, a mTORC1 target, depending on AKT activation. Notably, AKT inhibition resulted in ERK1/2 activation. Thus, the concomitant inhibition of ERK1/2 and AKT caused a stronger 4EBP1 de-phosphorylation and was required to induce a more efficient cytotoxicity.

**Abstract:**

mTOR is constitutively activated in acute myeloid leukemia (AML) cells, as indicated by the phosphorylation of its substrates, 4EBP1 and P70S6K. Here, we found that quercetin (Q) and rapamycin (Rap) inhibited P70S6K phosphorylation, partially dephosphorylated 4EBP1, and activated ERK1/2 in U937 and THP1, two leukemia cell lines. ERK1/2 inhibition by U0126 induced a stronger dephosphorylation of mTORC1 substrates and activated AKT. The concomitant inhibition of ERK1/2 and AKT further dephosphorylated 4EBP1 and further increased Q- or Rap-mediated cytotoxicity, compared to the single ERK1/2 or AKT inhibition in cells undergoing Q- or Rap-treatments. Moreover, quercetin or rapamycin reduced autophagy, particularly when used in combination with the ERK1/2 inhibitor, U0126. This effect was not dependent on TFEB localization in nuclei or cytoplasm or on the transcription of different autophagy genes, but did correlate with the reduction in protein translation due to a strong eIF2α-Ser51 phosphorylation. Thus, ERK1/2, by limiting 4EBP1 de-phosphorylation and eIF2α phosphorylation, behaves as a paladin of protein synthesis. Based on these findings, the combined inhibition of mTORC1, ERK1/2, and AKT should be considered in treatment of AML.

## 1. Introduction

The mammalian target of rapamycin (mTOR) is a conserved Ser/Thr kinase, entering in the formation of two molecular complexes, namely mTOR complex 1 (mTORC1) and mTOR complex 2 (mTORC2). mTOR maintains cell homeostasis through the regulation of many cellular functions, including transcription, translation, metabolism, autophagy, cytoskeletal organization, and cell survival [1,2,3,4,5]. mTORC1 is essential in regulating the cap-dependent translation initiation of many proteins through the phosphorylation of p70 ribosomal protein S6 kinase (P70S6K) and that of the inhibitor of protein synthesis eukaryotic translation initiation factor 4E (eIF4E) binding protein 1, known as 4EBP1 [6,7]. mTORC1 is primarily activated by hormones and growth factors that, by stimulating receptor tyrosine kinases (RTKs) such as insulin receptor kinases or IGF receptor kinases, activate phoshoinositide 3-kinase (PI3K). This results in phosphatidylinositol-3,4,5-triphosphate (PIP3) generation and the recruitment of phosphoinositide-dependent kinase 1 (PDK1) and AKT to the plasma membrane, where PDK1 activates AKT, via Thr308 phosphorylation [8]. AKT in turn phosphorylates tuberous sclerosis complex 2 (TSC2) [9,10], leading to dissociation of the TSC complex (TSC1/TSC2) from the lysosomal membrane and activation of RAS homolog enriched in brain (Rheb) [11,12], which directly binds and activates mTORC1 [13]. In addition to PI3K, mTORC1 may be activated by growth factors through RAS signaling [6,14] via the RAF/mitogen-activated protein kinase/extracellular signal-regulated kinase (RAF/MEK/ERK) axis and ERK effector p90-ribosomal S6 kinase, both phosphorylating and inhibiting TSC2 [15,16]. 

The mTOR axis is dysregulated in many cancer cells, thus representing a promising target in anti-cancer therapy. However, it has been shown that rapamycin (Rap) and its analogs (rapalogs), which are allosteric inhibitors of mTORC1, can cause activation of AKT. The relief of mTORC1/P70S6K-mediated feedback inhibition of RTKs or substrates, such as platelet-derived growth factor receptor (PDGFR) [17,18] and insulin receptor substrate-1 (IRS-1), has been shown to be the underlying mechanism involved [19,20]. Thus, the inhibition of the negative feedback loop leading to AKT activation represents a major drawback of treatments by rapalogs [21]. Furthermore, mTORC1 inhibition, by reducing the phosphorylation of P70S6K, was shown to induce the activation of RAS and MAPK/ERK [22]. 

RAS–RAF–MEK–ERK is another pathway that is often dysregulated in tumors, as a result of activating mutations in *RTK*, *RAS*, and *B-RAF* [23]. In AMLs, the most common type of acute leukemia, which is characterized by the lowest survival rate among all leukemia types, *RAS* is estimated to be mutated with a frequency of 15–40% ([24] and citations therein). Those mutations represent negative prognostic factors, correlating with poor survival in patients affected by AML [24]. It appears to be evident that a better understanding of mTORC1 and ERK activation and of the crosstalk between these pathways is desirable in order to reach a more effective targeted therapy and to improve the survival of AML patients.

In this work, we investigated the relationship between mTORC1 and ERK1/2 pathways in U937 and THP1, two AML cell lines, undergoing treatment by quercetin (3,3′,4′,5,7-pentahydroxyflavone) (Q) or Rap.

Q, a flavonoid widely distributed in plants and fruits such as apples, onions, and tea, is known for its antioxidant, anti-inflammatory, antiproliferative, chemo-preventive, and anti-carcinogenic properties [25,26]. The anti-cancer activity of this flavonoid has been reported to occur mainly through the inhibition of mTOR signaling [27,28]. However, this flavonoid has been suggested to have a hormetic nature (i.e., a biphasic dose–response relationship), as at low doses it shows an antioxidant activity, while at high doses it has a prooxidant property [29]. Its multitargeting effects have been reviewed [30] and its antiproliferative/apoptotic activities in vitro against AML cells, including U937 and THP1 cells, have been documented [31,32,33,34,35,36]. Here, we observed that Q or Rap, at doses close to those attainable in in vivo treatments, caused a partial inhibition of mTORC1 in U937 and in THP1 cells, as shown by the reduced phosphorylation of 4EBP1 at Ser65 and of P70S6K at Thr389. Concomitantly to the effect observed on mTORC1, Q and Rap activated ERK1/2 and decreased the autophagy flux. ERK1/2 inhibition further inhibited 4EBP1 and P70S6K phosphorylation and reduced autophagy, increasing cell death. The reduction of autophagy was not due to a defective transcription of autophagy genes, but was dependent on the inhibition of protein translation, which was even more evident when Q and Rap were combined with ERK inhibition. 

Different signaling cascades are responsible for the control of protein translation, including the PI3K/AKT/mTORC1 and ERK pathways, through 4EBP1 dephosphorylation and the phosphorylation of eukaryotic translation initiation factor 2 subunit 1 (eIF2α) at Ser51 [37,38,39]. Both effects occurred following ERK inhibition in combination with Q or Rap, suggesting that a general protein synthesis shutdown may be induced in AML cells undergoing such combined treatment.

## 2. Materials and Methods

### 2.1. Materials

Antibody anti-SQSTM1/p62 (BD Biosciences 610832) was purchased from BD Transduction Laboratories (San Jose, CA, USA). Antibodies purchased from Cell Signaling Technology (Danvers, MA, USA) were anti-4EBP1 (Cat# 9452), -phospho-ERK-Thr202/Tyr204 (Cat# 4370), -ERK (Cat# 4695), -phospho-AKT-Ser473 (Cat# 4060), -AKT (Cat# 4685), -Histone-H3 (Cat# 4499), -microtubule-associated protein 1A/1B-light chain 3 (LC3I/II) (Cat# 2775), -phospho-P70S6K-Thr389 (Cat# 9234), -P70S6K (Cat# 9202), phospho-eIF2α–Ser51 (Cat# 3398), eIF2α (Cat# 9722), -Transcription Factor EB (TFEB) (Cat# 4240), -Tubulin (Cat# 2146), horseradish peroxidase (HRP)-conjugated anti-rabbit-antibodies (Cat# 7074), and HRP-conjugated anti-mouse-antibodies (Cat# 7076). Anti-phospho-4EBP1-Ser65 (SC-293124) and radioimmunoprecipitation assay buffer (RIPA) were obtained from Santa Cruz Biotechnology Inc. (Dallas, TX, USA). Antibodies anti-β Actin (Cat# A1978), anti-Lamin B (Cat# 6216), and anti-Puromycin (clone 12D10) (Cat# MABE343), Bafilomycin A1 (Baf), bicinchoninic acid assay (BCA), bovine serum albumin (BSA), cycloheximide (CHX), fetal calf serum (FCS), Hanks’ balanced salt solution (HBSS), Ficoll–Hypaque, l-glutamine, penicillin-streptomycin, phosphate buffered saline (PBS), propidium iodide (PI), puromycin, quercetin, RNAse, RPMI-1640, U0126, and Wortmannin were obtained from Sigma–Aldrich (St. Louis, MO, USA). Rapamycin, PF-4708671, and MK2206 were obtained from Selleckchem (Houston, TX, USA). Reducing agent and detergent compatible (RC-DC) protein assay, sodium dodecyl sulfate (SDS)-sample buffer, protein standard, SDS-polyacrylamide gel electrophoresis (SDS-PAGE) reagents, polyvinylidene difluoride (PVDF), and nitrocellulose membranes were from Bio-Rad Laboratories (Segrate, Italy). LiteAblot TURBO chemiluminescence reagents were obtained from EuroClone (Pero, Milan, Italy). Lipofectamine RNAiMAX was obtained from Life Technologies. Other reagents of the highest purity were purchased from Bio-Rad or Sigma.

### 2.2. Cells and Viability

After obtaining ethic committee approval (Prot.847/19) and informed consent from all subjects involved in the study, peripheral blood mononuclear cells (PBMC) from healthy blood donors were obtained by Ficoll–Hypaque density blood centrifugation. U-937 cell line exhibiting monocyte morphology derived from patients with histiocytic lymphoma [40], THP1 acute monocytic leukemia [41], and HL-60, an acute promyleocytic leukemia cell line, were used. Cell were obtained from Prof. Teresa Fiorillo’s and Prof. Elisa Pietrangeli’s laboratories. U937, THP1, and HL-60 cells were grown in RPMI-1640 medium, 10% heat-inactivated FCS, 2 mM glutamine, 100 units/mL penicillin, and 100 μg/mL streptomycin, at 37 °C in fully humidified 95% room air/5% CO_2_. Cells were ≥93% viable, based on calculating alive trypan blue-excluding cells in terms of the percentage of all cells counted. Washed cells were resuspended in complete medium, 1 × 10^6^/mL, transferred to multi-well plates, treated with vehicles or inhibitors, incubated for 15 min, and subsequently exposed again to vehicles or to test agents. At the end of each experiment, aliquots of gently mixed cells were taken for PI staining and cell-cycle analysis. DMSO was used to solubilize drugs, except for puromycin, which was solubilized in H_2_O. The final amount of DMSO in the cultures was ≤0.3% (*v*/*v*), even when more drugs were used in combination. Mother solutions of the used drugs were Rap 5 mM, Q 100 mM, U0126 20 mM, PF-4708671 10 mM, Wortmannin 10 mM, Puromycin 10 mM, Bafilomycin 10 mM, and CHX 10 mM. Vehicle testing, even in the largest concentration, did not modify any investigated parameter in comparison with control cultures.

### 2.3. Flow Cytometry Analysis of Cell Death

Flow cytometry was used to evaluate nuclear DNA fragmentation as hypodiploic (subG1) DNA events after cell fixation and PI staining [42]. Briefly, washed cells were pelleted and fixed for 1h in ice cold ethanol/water (70/30, *v*/*v*), pelleted again, washed twice with PBS, and finally resuspended in PBS containing RNAse (20 μg/mL) and PI (0.1 μg/mL). Events in the different cell-cycle phases were gated using an EPICS XL cytofluorimeter (Beckman Coulter, Hialeah, FL, USA). At least 10,000 events in each sample were acquired. The data were analyzed using “Multicycle software for DNA content and cell cycle analysis” (Phoenix Flow System, San Diego, CA, USA). SubG1 events, representative of apoptotic events, were expressed as a percentage of total cell population. Membrane permeability, indicative of cell death, was evaluated by flow cytometry (EPICS XL) after cell resuspension in HBSS containing PI (20 μg/mL) at room temperature. Analysis of samples was carried out on an EPICS-XL flow cytometer (Coulter, Hialeah, FL, USA) equipped with an air-cooled argon laser tuned to 488 nm, measuring the fluorescence emission (FL3) at >575 nm. A 620 nm band pass filter was used to collect specific fluorescence signal corresponding to PI. A total of 10,000 cells were scored in each sample.

### 2.4. Western Blot Analysis

In order to obtain cell lysates, cells were kept on ice for 30 min, vortexed every 10 min in RIPA buffer, and supplemented with proteases and inhibitors of phosphatases; then, samples were cleared by 14,000× *g* centrifugation at 4 °C. An aliquot of the supernatant was used for protein quantification. Each sample was diluted to contain the same protein amount. Appropriate volumes of SDS-sample buffer (*v*/*v*) were added. Cell lysates were warmed at 95 °C for 5 min and cleared by 14,000× *g* centrifugation in a microfuge for 15 min at 4 °C. Equal protein amounts were separated from different samples by SDS–PAGE and transferred onto PVDF membranes. Red Ponceau staining was used to check transfer efficiency. Blots were blocked in Tris-buffered saline, Tween-20 0.1% (*v*/*v*) (TBS-T), containing BSA 5% (*w*/*v*); they were probed with specific primary antibodies, washed with TBS-T, and incubated with HRP-conjugated antibodies. For each antibody, the dilutions and incubation times suggested by the manufacturer were used. To control protein loading, the product of a housekeeping gene was probed in each membrane. Membranes with cytosol and nuclear proteins were probed with antibodies anti β-Actin, anti-Histone H3, and anti Lamin B. Immunodetection was performed using LiteAblot chemiluminescent reagents. Densitometry quantitation of bands was performed by a Mac OS X (Apple Computer International, Cupertino, CA, USA) using ImageJ software (National Institutes of Health, Bethesda, MD, USA).

### 2.5. Protein Synthesis Assay—Surface Sensing of Translation (SUnSET) [43]

At 30 min from the end of the experiments, puromycin (10 μg/mL) was added to the cells. Then, as indicated above, the cells were kept for 30 min on ice in lysis buffer, and protein extraction and quantitation were performed. Appropriate volumes of unreducing SDS-sample buffer (*v*/*v*) were added to each sample. Cell lysates were warmed at 95 °C for 5 min and cleared by 14,000× *g* centrifugation for 15 min at 4 °C. Equal protein amounts were separated from each sample by SDS–PAGE and blotted onto nitrocellulose membranes. Transfer efficiency was checked by red Ponceau staining. Blots were blocked with 5% (*w*/*v*) milk in TBS-T at room temperature for 1 h and incubated overnight at 4 °C with mouse anti-Puromycin, clone 12D10, diluted 1:25,000. Then, membranes were washed three times for 15 min with TBS-T and incubated for 1 h at room temperature with HRP-conjugated goat anti-mouse IgG. Puromycin-labeled proteins were detected using LiteAblot chemiluminescent reagents. 

### 2.6. Silencing of ERK1/2

RNA knockdown was performed with pools of siRNA duplexes. Washed cells were resuspended in OPTI-MEM medium, transfected with siRNA specific for p44/42 MAPK (Cell Signaling, Cat# 6530) or with scrambled siRNA (Cell Signaling, Cat# 6568), using Lipofectamine RNAiMAX according to the guidelines of the manufacturer. RPMI 1640, 20% FCS, was added after 12 h, without the removal of the transfection medium. The cells were cultured for a further 60 h. After centrifugation, the medium was replaced with RPMI 1640, 10% FCS, and the cells were treated with Q, or not treated.

### 2.7. Real-Time Quantitative Polymerase Chain Reaction (RT-qPCR)

Analysis of Beclin (BECN1) and TFEB target gene mRNA levels (Map1LC3b, SQSTM1/p62, ULK1 and LAMP1) was performed using RT-qPCR. Total RNA was isolated by TRIzol reagent and reverse transcribed using a cDNA Reverse Transcription kit. Relative RT-qPCR was performed in a total reaction volume of 20 μL, using 2 μL of cDNA, 1 μL of primer forward, 1 μL of primer reverse, 10 μL of SYBR Green qPCR Master Mix, and 6 μL H_2_O. Amplification conditions were as follows: initial denaturation at 95 °C for 15 min, followed by 40 cycles comprising denaturation at 95 °C for 10 s, annealing at 60 °C for 20 s, and extension at 72 °C for 30 s. Cycle threshold (*Ct*) values, corresponding to the PCR cycle number at which the fluorescence emission reaches a threshold above the baseline emission, was determined and the mRNA expression values were calculated using β2-Microglobulin as the endogenous control according to the comparative *Ct* (ΔΔ*Ct*) method. Primers were purchased from Bio-Fab research (Bio-Fab research srl, Rome, Italy) and designed as shown in Table 1:

### 2.8. Subcellular Fractionation

U937 cells were collected by centrifugation at 600× *g* for 5 min at 4 °C and lysed using the Nuclear/Cytosol Fractionation Kit from BioVision (Milpitas, CA, USA), according to the manufacturer’s instructions. Briefly, cells, washed with PBS, were lysed in the cytosol extraction buffer A containing DTT and protease inhibitors, vortexed for 15 s, and incubated on ice for 10 min. Then, cytosol extraction buffer B was added to the lysates, which were incubated on ice for 1 min and centrifugated at 16,000× *g* for 5 min at 4 °C. Supernatants corresponding to cytoplasmic fractions were collected, while pellets were resuspended in nuclear extraction buffer mix containing DL-Dithiothreitol (DTT) and cocktail inhibitors. Each sample was vortexed for 15 s and incubated for 10 min on ice. This last step was repeated four times every 10 min. Finally, samples containing nuclear proteins were centrifuged at 16,000× *g* for 10 min at 4 °C and supernatants corresponding to nuclear fraction were collected. The detection of Histone H3, β-Actin, and Lamin B was used to evaluate subcellular fractions.

### 2.9. Analysis of the Western Blot Bands

The densitometry values of the bands were used to calculate the following ratio: (investigated protein/loading protein control in the same lysate)/(investigated protein in the lysate of untreated cells/loading protein control in the lysate of untreated cells).

The value under each band was obtained by calculating the ratio of the densitometry results in the shown blot, as detailed above.

### 2.10. Statistical Analysis

The results were expressed as mean ± SD of at least three independent experiments. Data were analyzed using one-way ANOVA for comparisons among groups. A *p*-value was considered statistically not significant (NS) when *p* ≥ 0.05, significant when *p* < 0.05, and very significant when *p* ≤ 0.01.

## 3. Results

### 3.1. mTORC1/P70S6K Inhibition Promotes ERK1/2 Activation in AML Cells

**Figure 1:**
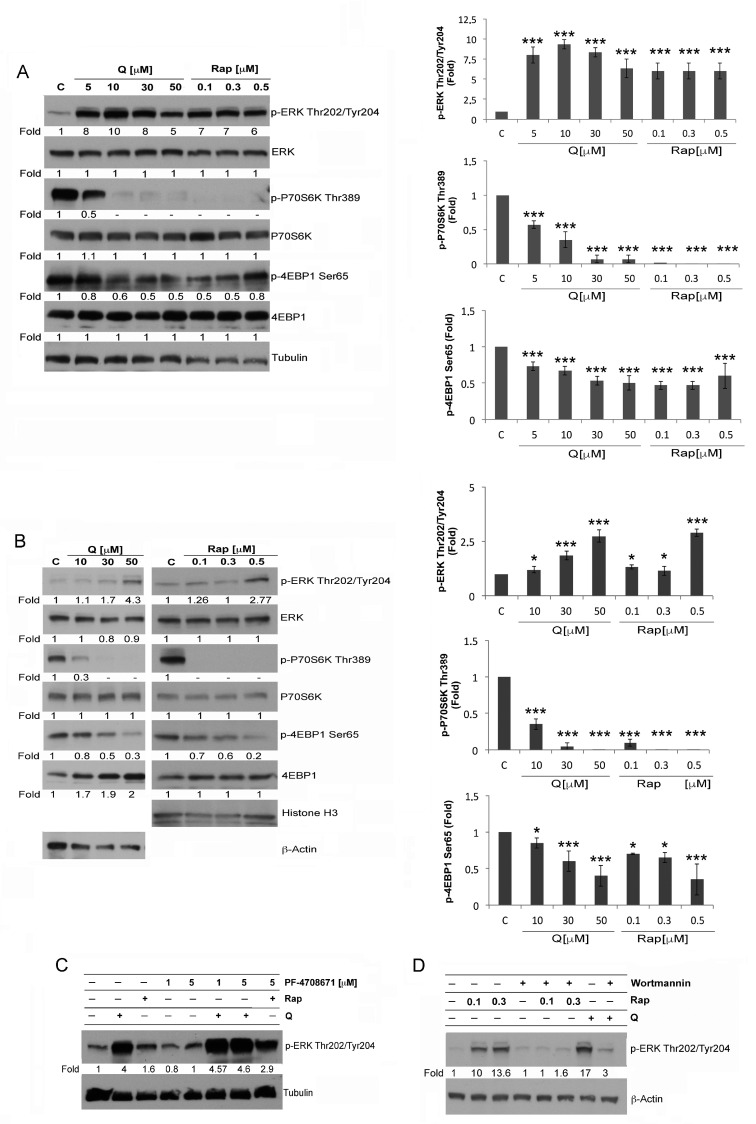
Inhibition of mTORC1 leads to ERK1/2 activation in AML cells. (**A**,**B**) Western blot analysis of phosphorylated and total amounts of ERK1/2, P70S6K, and 4EBP1 in U937 cells (**A**) and THP1 cells (**B**), treated or not for 6 h with the indicated concentrations of Rap or Q. The shown blots in A and B are representative, respectively, of four and three independent experiments. The densitometric values of the bands, obtained as described in the Materials and Methods section in four and in three independent experiments in U937 cells and in THP1 cells, respectively, were used to calculate the mean ± SD in the histograms, where * = *p* > 0.05 and *** = *p* ≤ 0.01 for comparison with the control group. (**C**) Western blot analysis of p-ERK in lysates of U937 cells pretreated or not with PF-4708671 (1 μM or 5 μM) for 15 min and, then, treated or not for 6 h with Q 10 μM or Rap 0.1 μM. The shown blot is representative of three independent experiments. (**D**) Western blot analysis of p-ERK in lysates of U937 cells pretreated or not with Wortmannin 10 μM for 15 min and, then, treated or not with Q 10 μM or Rap 0.1 μM for 6 h. The shown blot in D is representative of three independent experiments. Tubulin, β-Actin, and Histone H3 are shown as the control of protein loading. The value under each band was obtained as described in the Materials and Methods section.

mTORC1 signaling represents one of the most important pro-survival pathways in AML [44]. However, mTORC1 has been shown to inhibit ERK1/2 in different tumor types [22]. Thus, it appears important to explore the crosstalk between mTORC1 and ERK1/2 in AML cells. To this end, we evaluated the phosphorylation of ERK1/2 at Thr202/Tyr204 and of mTORC1 substrates, 4EBP1 at Ser65 and P70S6K at Thr389, in U937 and in THP1 cells treated by Q or Rap (Figure 1A,B) (Supplementary Materials). We performed dose–response experiments with Q or Rap and found an increase of ERK1/2 phosphorylation concomitantly with a de-phosphorylation of P70S6K in U937 cells (Q 10 μM, Rap 0.1 μM) (Figure 1A). Similar effects were observed in THP1 cells, although these cells were less sensitive and higher doses of Q (50 μM) or Rap (0.5 μM) were required to induce the above-reported effects (Figure 1B). This may be due to intrinsic differences in biochemical and molecular properties and/or in the maturation stage of this cell line. Interestingly, at different concentrations, Q or Rap caused a partial inhibition of 4EBP1 phosphorylation at Ser65 in both cell lines (Figure 1A,B).

Next, we examined the impact of P70S6K inhibition on ERK1/2 phosphorylation and found that such inhibition by PF-4708671 [45] further increased the ERK1/2 activation caused by Q or Rap (Figure 1C). In contrast to pretreatment with PF-4708671, pretreatment with Wortmannin, a PI3K inhibitor, prevented ERK1/2 activation induced by Rap or Q treatments (Figure 1D). 

The differences observed on ERK1/2, P70S6K, and 4EBP1 occurred at the phosphorylation level, as the total amounts of these proteins were not affected by these treatments (Figure 1A–D). 

These data indicate that mTORC1 negatively regulates MEK/ERK and, therefore, mTORC1 inhibition results in ERK1/2 activation in AML cells.

### 3.2. ERK1/2, Retrived from mTORC1 Dependent Inhibition, Maintains mTORC1 Activation

**Figure 2:**
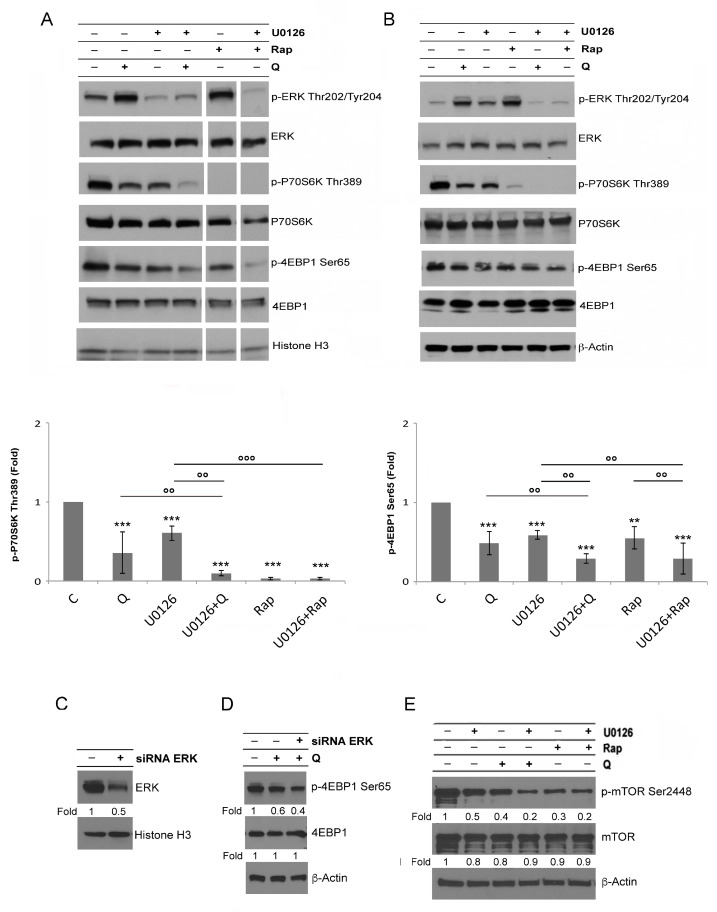
ERK1/2 counteracts mTORC1 inhibition in AML cells. (**A**) Western blot analysis of phosphorylated and total amounts of ERK, P70S6K, and 4EBP1 in lysates of U937 cells pretreated or not with U0126 (20 μM for 15 min) and, then, treated or not for 6 h with Q 10 μM or Rap 0.1 μM. (**B**) Western blot analysis of phosphorylated and total amounts of ERK, P70S6K, and 4EBP1 in lysates of THP1 cells pretreated or not with U0126 (20 μM for 15 min) and, then, treated or not for 6 h with Q 50 μM or Rap 0.5 μM. The densitometric values of the bands, obtained as described in the Materials and Methods section in three independent experiments, were used to calculate mean ± SD in the histograms, where ** = *p* < 0.05 and *** = *p* ≤ 0.01 for comparison with the control group and °° = *p* < 0.05 and °°° = *p* ≤ 0.01 for comparison with the indicated groups. (**C**) ERK1/2 silenced U937 cells were treated with (**D**) Q 10 μM and the impact on 4EBP1 phosphorylation was evaluated by Western blot. (**E**) Western blot analysis of mTOR phosphorylation in Ser2448 in lysates of U937 cells pretreated or not with U0126 (20 μM for 15 min) and, then, treated or not for 6 h with Q 10 μM or Rap 0.1 μM. (**A**–**E**) β-Actin and Histone H3 are shown as the control of protein loading. (**C**–**E**) The value under each band was obtained as described in the Materials and Methods section.

We next investigated the effect of ERK1/2 on mTORC1 and found that this MAPK, retrieved from the mTORC1 dependent inhibitory effect, limited mTORC1 inhibition in Rap- and Q-treated U937 and THP1 cells. In fact, the pharmacological inhibition of ERK1/2 by U0126 (20 μM, 15min pre-treatment), which moderately decreased the constitutive phosphorylation of P70S6K and 4EBP1, caused a stronger inhibition of these mTORC1 targets in combination with Rap or Q (Figure 2A,B). Similar results were obtained by performing ERK1/2 silencing before cell exposure to Q (Figure 2C,D). mTOR, constitutively phosphorylated in Ser2448 in U937 cells, was partially inhibited by Q 10 μM, Rap 0.1 μM, or U0126 20 μM and to a larger extent dephosphorylated by combination treatments with U0126 + Q or U0126 + Rap (Figure 2E). These results indicate that ERK1/2, retrieved from the mTORC1 inhibitory effect, positively regulates mTORC1 activation.

**Figure 3:**
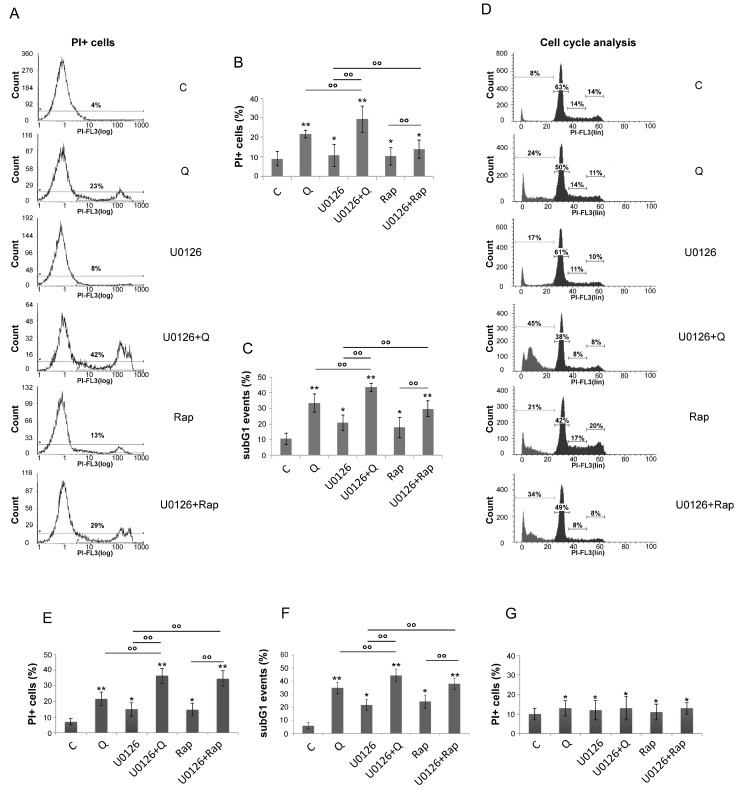
ERK1/2 contributes to cell survival. Cell death was investigated in U937 cells (**A**–**D**), HL-60 cells (**E**,**F**), or PBMC cells (**G**) pretreated or not with U0126 (20 μM for 15 min) and, then, treated or not for 24 h with Q 10 μM or Rap 0.1 μM by calculating PI+ cells as a percentage of total cells examined by cytofluorimetry (**A**,**B**,**E**,**G**) and after cell fixation and staining with PI in order to detect subG1 events in the cell cycle by cytofluorimetry (**C**,**D**,**F**). For each parameter ≥10,000 events were examined in each sample. The shown values represent mean ± SD of three independent experiments (**B**,**C**,**E**,**F**,**G**). * = *p* ≥ 0.05 and ** = *p* < 0.05 for comparison with the control group; °° = *p* < 0.05 for comparison with the indicated groups. Results obtained in a single experiment are also shown (**A**,**D**).

ERK1/2 and mTORC1 are central players in cancer survival. Thus, we sought to investigate their role in the cell survival of AML cells. Q or Rap induced a mild cytotoxic effect in U937 cells, as Q caused an increase of PI + cells (21 ± 2.1% vs. 9 ± 3.6%) and of subG1 events (33 ± 5.8% vs. 10 ± 3.6%) (Figure 3A–D). ERK1/2 inhibition was per se slightly cytotoxic (10.6 ± 5.7% PI+ cells and 20 ± 4.7% subG1 events) but it strongly potentiated Q-induced cytotoxicity (29 ± 6.8% PI+ cells and 43 ± 2.5% subG1 events) (Figure 3A–D). The same occurred for Rap, which caused 10.3 ± 4.5% PI+ cells and 17.7 ± 6.4% subG1 events, while in combination with ERK1/2 inhibition its cytotoxicity increased, particularly in terms of subG1 events (30 ± 5%) (Figure 3A–D). In addition, HL-60 cells were more efficiently killed when mTORC1 and ERK were concomitantly inhibited (Figure 3E,F), while PBMC, obtained from healthy blood donors and treated similarly to AML cells, was unaffected by those treatments (Figure 3G).

These results suggest that ERK1/2, retrieved from mTORC1 inhibition, protected mTORC1 from excessive de-phosphorylation and played a pro-survival role in AML cells. 

**Figure 4:**
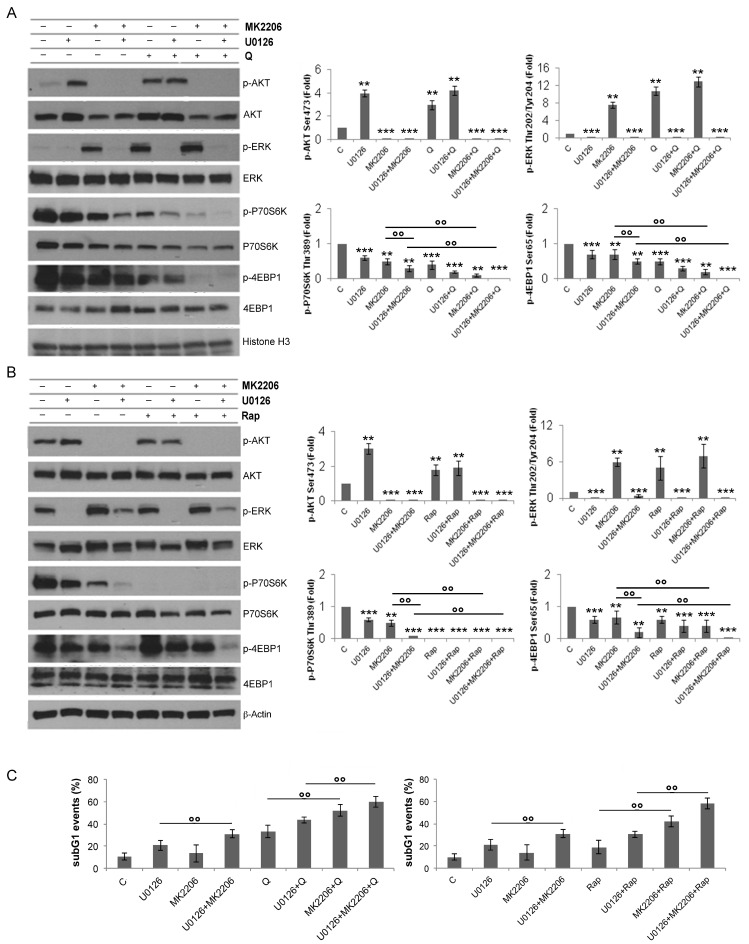
ERK1/2 inhibition is accompanied by AKT activation and the concomitant inhibition results in a further dephosphorylation of 4EBP1. (**A**,**B**) Western blot analysis of p-AKT, AKT, p-ERK, ERK, p-P70S6K, p-4EBP1, and 4EBP1 in lysates of U937 cells pretreated or not with U0126 20 μM, MK2206 1 μM, and U0126 + MK2206 for 15 min and, then, treated or not for 6 h with Q 10 μM (**A**) or Rap 0.1 μM (**B**). The densitometric values of the bands, obtained as described in the Materials and Methods section, in three independent experiments in U937 cells were used to calculate the mean ± SD in the histograms, where ** = *p* < 0.05 and *** = *p* ≤ 0.01 for comparison with the control group and °° = *p* < 0.05 for comparison with the indicated groups. (**C**) Cell death was investigated in U937 cells, treated for 24 h, as indicated above, after cell fixation and staining with PI in order to detect subG1 events in the cell cycle by cytofluorimetry. ≥10,000 events were examined in each sample. The shown values represent the mean ± SD of three independent experiments. °° = *p* < 0.05 for comparison of the indicated groups.

We then found that ERK1/2 inhibition by U0126 was accompanied by AKT activation (Figure 4A,B). The concomitant inhibition of ERK1/2 by U0126 (20 μM) and AKT by MK2206 (1 μM) further dephosphorylated 4EBP1 in cells treated by Q or Rap (Figure 4A,B) and, accordingly, further increased Q- or Rap-mediated cytotoxicity, compared to the single ERK1/2 or AKT inhibition (Figure 4C). Of interest, AKT inhibition by MK2206 (1 μM) was accompanied by ERK activation (Figure 4A,B), indicating a mutual crosstalk between ERK and AKT.

### 3.3. ERK1/2 Counteracts Q- or Rap-Mediated Autophagy Inhibition

**Figure 5:**
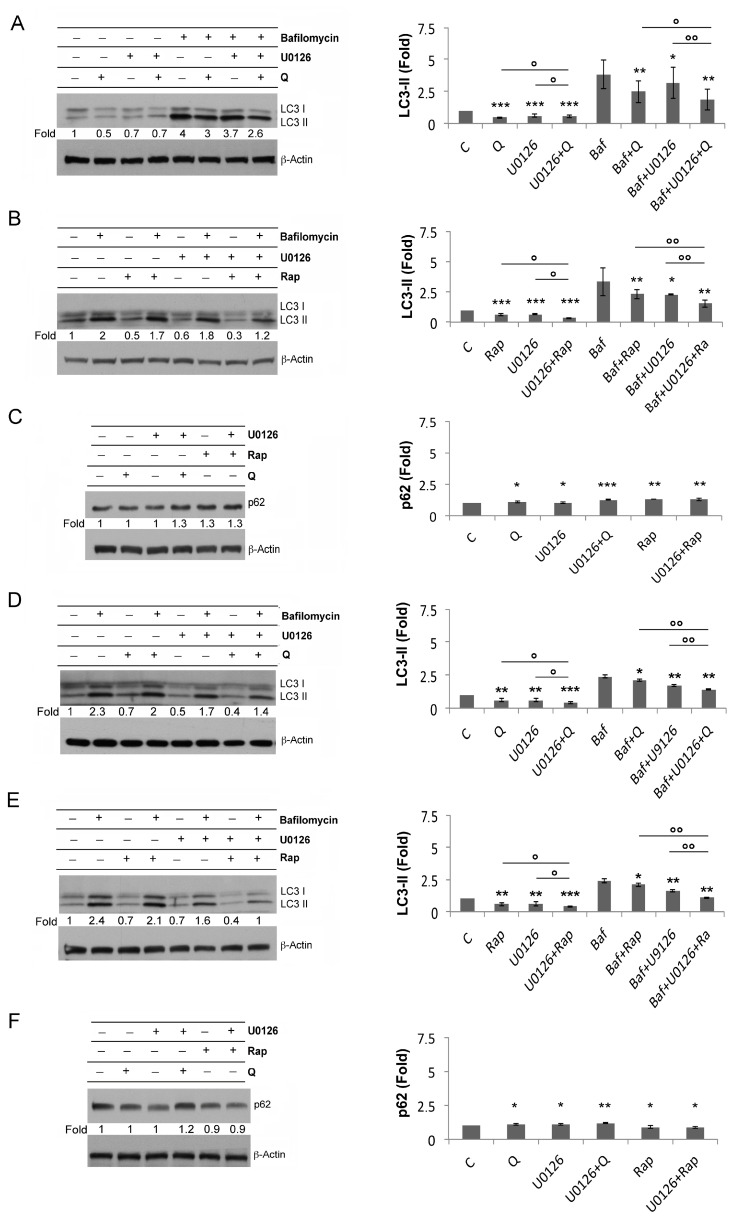
ERK1/2 contrasts autophagy reduction induced by Q or Rap. (**A**–**C**) Western blot analysis of LC3-II or p62 in U937 cells pretreated or not with U0126 (20 μM for 15 min) and, then, treated or not for 6 h with Q 10 μM (**A**,**C**) or with Rap 0.1 μM (**B**,**C**). In (**A**,**B**) the same samples were treated side-by-side during the last 3 h also with Baf (10 μM). (**A**–**C**) Densitometric analysis of LC3-II and p62 after the Western blot of U937 cells pretreated or not with U0126 (20 μM, for 15 min) and, then, treated or not for 6 h with Q 10 μM or Rap 0.1 μM. The densitometric values (mean ± SD) were obtained by three independent experiments in U937 cells, where * = *p* ≥ 0.05 (NS), ** = *p* < 0.05, and *** = *p* ≤ 0.01 for comparison with the control groups (either untreated or Baf treated). ° = *p* ≥ 0.05 (NS) °° = *p* < 0.05 for comparison with the indicated groups. (**D**–**F**) Western blot analysis of LC3-II or p62 in THP1 cells pretreated or not with U0126 (20 μM for 15 min) and, then, treated or not for 6 h with Q 50 μM or with Rap 0.5 μM. In (**D**,**E**), the same samples were treated side-by-side, also with Baf (10 μM), for the last 3 h. The densitometric values of the bands, obtained as described in the Materials and Methods section, in three independent experiments in THP1 cells were used to calculate the mean ± SD in the histograms, where * = *p* ≥ 0.05 (NS), ** = *p* < 0.05, and *** = *p* ≤ 0.01 for comparison with either the untreated or the Baf-treated control group. ° = *p* ≥ 0.05 (NS) and °° = *p* < 0.05, for comparison with the indicated groups. β-Actin was probed to control the protein load among the samples.

Autophagy is an important catabolic process that is constitutively activated in cancer cells, by which they degrade damaged components and maintain bioenergetic levels. However, excessive or defective autophagy may promote cancer cell death. Conditions of mTORC1 inhibition are known to activate the autophagy flux [46]. To explore autophagy, in this study, we analyzed LC3-II by Western blot in AML cells pretreated or not with U0126 and, then, treated by Q or with Rap in the presence of or in the absence of Bafilomycin A1 (Baf), which was added during the last 3 h. The latter blocks the degradative steps of autophagy and, as a consequence, allows the evaluation of LC3-II formation [47]. The LC3-II expression level was rather low in untreated U937 cells (1×) and after Q treatment (×0.5); however, Baf increased the LC3-II accumulation in the control cells (×4) and, to lesser extent, in Q-treated cells (×3) (Figure 5A). ERK inhibition by U0126 partially reduced LC3-II (×0.7), also in the presence of Baf (×3.7), while the combination of U0126 + Q in the presence of Baf induced a larger decrease of LC3-II (×2.6) (Figure 5A). Similar results were obtained by using Rap instead of Q in combination with U0126 (Figure 5B). Furthermore, we analyzed SQSTM1/p62 by Western blot, as this protein, being degraded during the final autophagy steps, represents another readout of autophagy flux [47]. We found that the amount of this protein was unaffected by Q (1×) or U0126 (1×) in comparison to untreated U937 cells (1×), while it increased when these cells were treated with U0126 + Q (×1.3), Rap (×1.3), or U0126 + Rap (×1.3) (Figure 5C). The autophagy flux was also investigated in THP1 cells and we obtained similar results (Figure 5D–F). 

Altogether, these findings suggest that Rap or Q reduced the autophagy flux, particularly in combination with ERK1/2 inhibition, despite inducing a stronger inhibition of mTORC1. 

### 3.4. Autophagy Reduction Is Not Caused by Transcriptional Inhibition of Autophagy Genes

**Figure 6:**
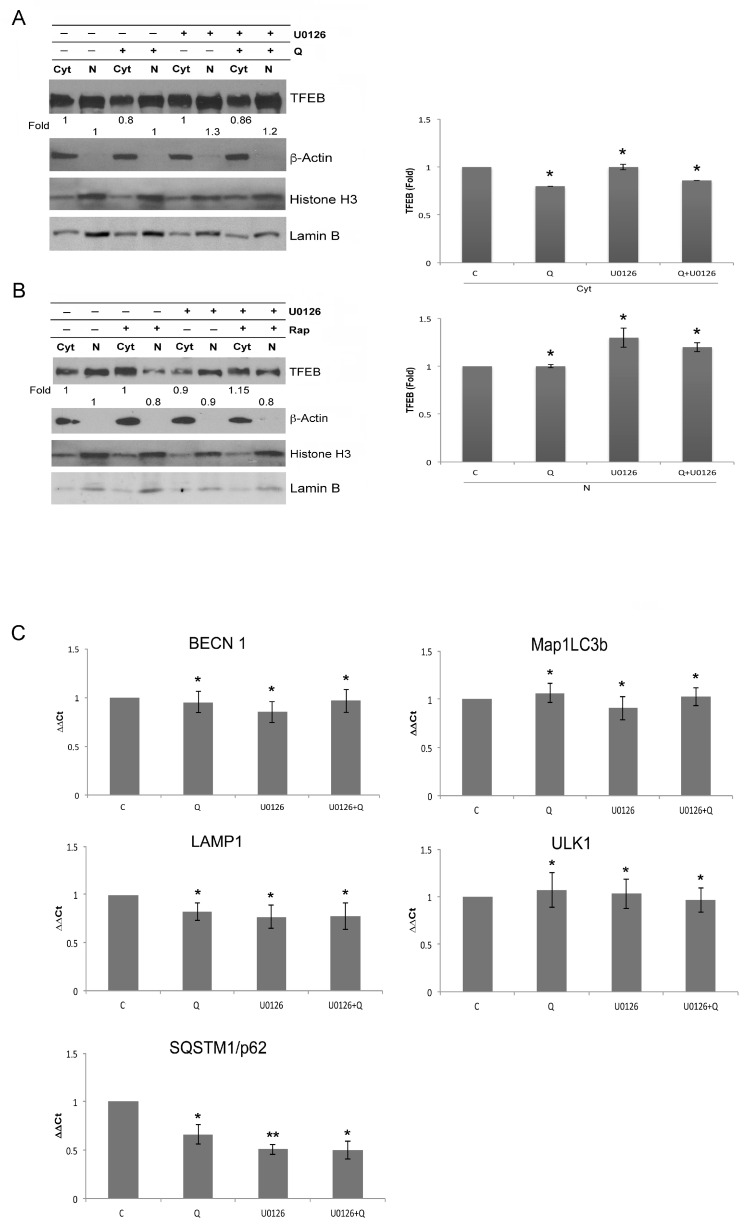
TFEB compartmentalization and transcription of autophagy genes are not dependent on ERK1/2. (**A**,**B**) Western blot analysis of TFEB in cytosol or nuclear lysates of U937 cells pretreated or not with U0126 (20 μM for 15 min) and treated or not for 6 h with Q 10 μM or Rap 0.1 μM. anti β-Actin, anti-Histone H3, and anti-Lamin B, were used as the loading control. Values under each band relative to cytosol lysates (Cyt) were obtained using the following formula: (densitometry value of TFEB band in Cyt lysate under examination/densitometry value of β-Actin band in the same Cyt lysate)/(densitometry value of TFEB band in Cyt lysate of untreated cells/densitometry value of β-Actin band in Cyt lysate of untreated cells). The values under each band relative to nuclear lysates (N) were obtained using the following formula: (densitometry value of TFEB band in N lysate under examination/densitometry value of Histone H3 band in the same N lysate)/(densitometry value of TFEB band in N lysate of untreated cells/densitometry value of Histone H3 band in the N lysate of untreated cells). The shown blots are representative of three independent experiments. The densitometric values of the bands, obtained as described above and in the Materials and Methods section, in three independent experiments in U937 cells were used to calculate the mean ± SD in the histograms, where * = *p* ≥ 0.05 (NS) for comparison with the control group. (**C**) Quantitative RT-PCR of BECN1, SQSTM1/p62, Map1LC3b, ULK1, and LAMP1 mRNA in U937 cells pretreated or not with U0126 (20 μM for 15 min) and treated or not for 6 h with Q 10 μM. The values represent the mean ± SD of three independent experiments. * = *p* ≥ 0.05 (NS) and ** = *p* < 0.05 for comparison with the control group.

TFEB is a master transcription factor that coordinates the transcription of a gene network that controls the formation of autophagosomes, autophagosome-lysosome fusion, lysosome function, and biogenesis. In the unphosphorylated state, it migrates to the nucleus and induces the expression of autophagy and lysosome genes [48,49,50,51,52]. TFEB phosphorylation and its subcellular localization are under the control of activated mTORC1 and ERK1/2 [49,53]. Since Rap and Q induced mTORC1 inhibition and ERK1/2 activation, they could influence TFEB in an opposite way. Therefore, we investigated TFEB compartmentalization in U937 cells, pretreated or not with U0126 and, thereafter, exposed to Rap or Q. We found that these different treatments slightly modified the TFEB distribution between the nuclei and the cytoplasm, in comparison with untreated cells (Figure 6A,B). These conclusions were also drawn by considering a very low presence of β-Actin in the nuclear compartments and a low contamination of Histone H3 and Lamin B in the cytosol compartments. These data suggest that the reduction of the autophagy flux, as shown above, did not correlate with a reduced level of TFEB nuclear localization. However, since other transcription factors could be involved in the transcription of autophagic genes, we performed quantitative PCR on U937 cells treated by Q, U0126, and U0126 + Q. These treatments induced a slight decrease in the transcripts of SQSTM1/p62 in comparison to untreated cells, while they did not influence the transcription of Beclin-1, Map1LC3b, LAMP1, or ULK1 (Figure 6C).

Thus, the reduced autophagy flux was not due to decrease in the transcription of the investigated autophagy genes or in Q-treated cells, in which mTORC1 was partially inhibited and ERK1/2 was activated, or in cells in which ERK1/2 was inhibited and mTORC1 substrates were more strongly inhibited.

### 3.5. Activation of ERK1/2 Prevents Protein Synthesis Shutdown

**Figure 7:**
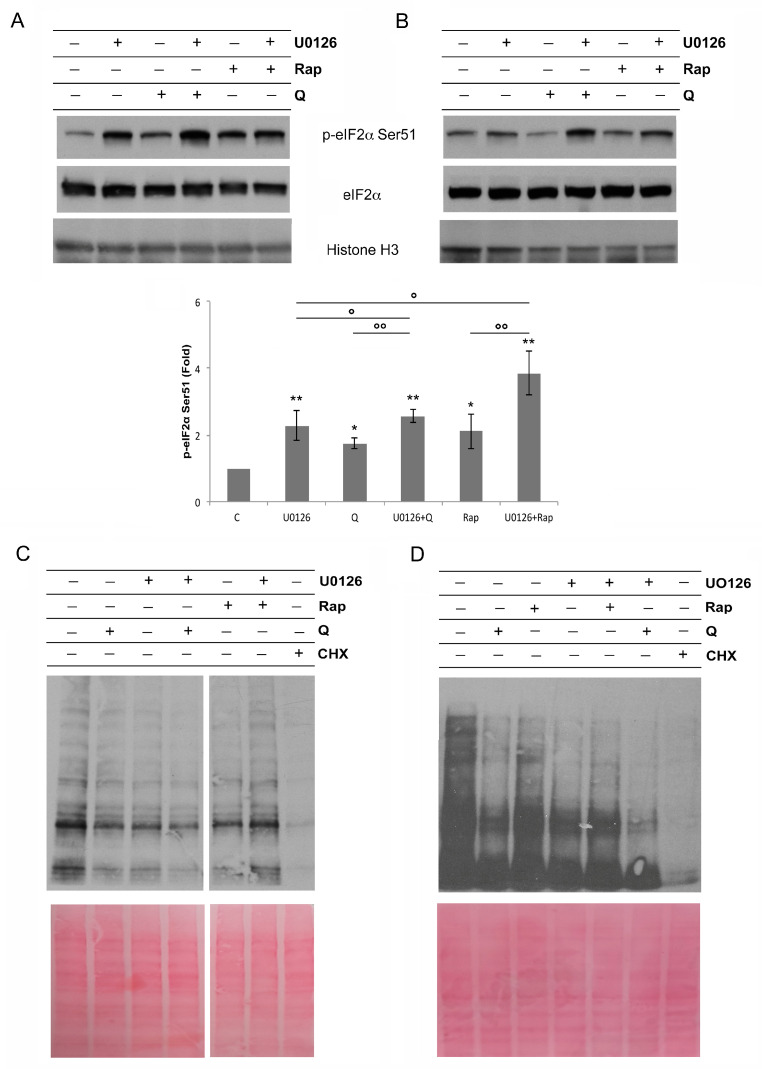
ERK1/2 contrasts p-eIF2α phosphorylation and protein synthesis inhibition. (**A**,**B**) Western blot analysis of p-eIF2α-Ser51 and eIF2α in U937 cells pretreated or not with U0126 (20 μM for 15 min) and, then, treated or not for 6 h with Q 10 μM or Rap 0.1 μM and in THP1 cells pretreated or not with U0126 (20 μM for 15 min) and, then, treated or not for 6 h with Q 50 μM or Rap 0.5 μM. Histone H3 was probed to control the protein load among the samples. The densitometric values of the bands, obtained as described in the Materials and Methods section in three independent experiments, in U937 cells were used to calculate the mean ± SD in the histograms, where * = *p* ≥ 0.05 (NS) and ** = *p* < 0.05 for comparison with the control group and ° = *p* ≥ 0.05 (NS) and °° *= p* < 0.05 for comparison with the indicated groups. (**C**,**D**) SUnSET analysis of protein synthesis by evaluation of puromycin bound to nascent proteins by specific mAb and HRP-conjugated antibodies in lysates of U937 cells or THP1 cells pretreated or not with U0126 (20 μM for 15 min) and, then, treated or not for 6 h with Q 10 μM or Rap 0.1 μ, in the case of U937 cells or with Q 50 μM and Rap 0.5 μM in the case of THP1 cells. Puromycin (10 μM) was added to the cells during the last 30 min of the experiment. U937 or THP1 cells treated with cycloheximide (CHX, 10 μM) for 6 h and puromycin (10 μM) in the last 15 min of the experiment were used as the control of the inhibition of protein synthesis (**C**,**D**). Membranes in the lower portion of (**C**,**D**) are the same membranes as in the upper portion and were stained with red Ponceau to control the protein load. The shown results were obtained in three and two independent experiments, respectively, with U937 cells and THP1 cells.

The above results suggested that in Rap- and Q-treated cells, the dephosphorylation of mTORC1 substrates inhibited protein translation, leading to a decrease in autophagy-relevant proteins and, therefore, reducing autophagy. As not only dephosphorylated 4EBP1, but also eIF2α phosphorylated at Ser51, may block protein synthesis at the translational level, we next explored the activation of eIF2α in U937 and in THP1 cells and found that U0126 caused its phosphorylation at Ser51, particularly when combined with Rap or Q (Figure 7A,B). Then, we monitored protein synthesis by immunoblots of protein extracts of U937 cells (Figure 7C) or THP1 cells (Figure 7D) pretreated or not with U0126 and, thereafter, exposed to Rap or Q, then treated with puromycin. Puromycin is a structural analog of aminoacyl-transfer RNA that is incorporated into nascent polypeptide chains, causing premature termination. Its incorporation in newly synthesized proteins can be detected using anti-puromycin antibodies [43]. In comparison to untreated cells, Rap-, Q- or U0126-treated cells showed a decreased labeling of bands probed with anti-puromycin antibodies. This suggests a reduction in protein synthesis. This effect was stronger following combined treatments with U0126 + Rap or U0126 + Q (Figure 7C,D). In Western blots, lysates of cells treated with cycloheximide were also included as a control of protein synthesis inhibition, detected by the SUnSET method (Figure 7C,D). In addition, in the lower portion of Figure 7C,D, we reported the same membranes used for the Western blot soon after red Ponceau staining, as the control of protein loading.

The above data show that the inhibition of mTORC1, leading to a protein synthesis decrease, was limited by ERK1/2 activation, and that this MAPK acted a as paladin of protein synthesis, counteracting 4EBP1 dephosphorylation and eIF2α phosphorylation.

## 4. Discussion

The aberrant activation of PI3K/AKT/mTOR signaling is a common finding in cancer, in which it plays a pro-survival role. The constitutive activation of PI3K/AKT/mTOR has been reported in 50–80% of AML patients, in correlation with a decrease in overall survival [54]; thus, key components of this pathway have been considered as possible targets for AML treatment. However, the use of PI3K/AKT/mTOR inhibitors as monotherapy has shown limited efficacy in clinical studies, due to the activation of pro-survival pathways [55]. It appears that better knowledge of the crosstalk between the different pro-survival pathways may help in the design of combination therapies that are able to treat AML more successfully.

The first aspect investigated in this study was the evaluation of whether mTORC1 inhibition could remove the inhibitory loop on ERK1/2 in AML cell lines, as shown by Carracedo et al. with respect to other tumor types [22]. The findings reported here showed that an inhibitory effect of mTORC1 on ERK1/2 also occurs in AML cells. It was removed by Rap, Q, and, to a larger extent, by the combination of them with the inhibitor of P70S6K, PF-4708671. 

Then, we investigated the influence of ERK1/2 on mTORC1 in our setting, as it is known that ERK1/2 contributes to the activation of mTORC1 [56,57,58]. According to previous results, MEK/ERK inhibition by U0126 led to a decrease in the constitutive activity of mTORC1 on its targets, P70S6K and 4EBP1, and a stronger inhibition of them was observed by using U0126 in combination with Rap or Q. Taken together, these results suggest that mTORC1, through P70S6K, limits the activation of ERK1/2 and that, on the other hand, this MAPK sustains the activity of mTORC1. As a consequence of this two-way loop, the inhibition of mTORC1 by Rap or Q could not be complete and cell survival was not strongly inhibited. However, this effect could be induced by the concomitant use of ERK1/2 and mTORC1, although some cells were still resistant to the combined treatment. These results suggest that multiple survival pathways orchestrated cell fate following mTORC1 inhibition. Indeed, this treatment also resulted in AKT activation, to an even larger extent, after inhibition of ERK. As a consequence, the concomitant inhibition of ERK and AKT was needed to obtain a strongly cytotoxic result.

In the present work, we also investigated the impact of mTORC1 and ERK1/2 inhibition on the autophagy flux, as this mechanism may contribute to cell survival. mTORC1, in the presence of sufficient nutrients and growth factors, upregulates anabolic processes, including protein, lipid, and nucleotide synthesis, while it inhibits catabolic pathways, including autophagy. U937 and THP1 cells showed basal autophagy flux in the presence of the constitutive activation of mTORC1, as indicated by the phosphorylation of mTORC1 targets, P70S6K, and 4EBP1. It seems that these cells “use” mTORC1 activity to sustain the autophagy flux. Q and Rap, although causing decreased mTORC1 activity, caused a reduction of the autophagy flux, as evidenced by the expression levels of SQSTM1/p62 and LC3-II. These findings were rather surprising—particularly those obtained with Rap, as this drug is generally used to inhibit mTORC1 and, therefore, to induce autophagy. However, it should also be considered that Rap has been previously reported to attenuate autophagy induced by 6-thioguanine in colorectal cancer cells [59], due to the removal of an mTOR-P70S6K inhibitory loop on AKT, a known inhibitor of autophagy [60]. Previous investigations have also shown that P70S6K is needed for starvation-induced autophagy in *Drosophila* body fat [61] and that the inhibition of mammalian S6 kinase by resveratrol suppresses autophagy [62]. Thus, in future studies, it will be interesting to investigate whether the decrease in P70S6K phosphorylation observed in this study could remove the inhibitory loop on AKT and whether the increase in AKT activation could contribute to the observed autophagy slowdown.

Here, we also examined the compartmentalization of TFEB between the nuclei and the cytoplasm, as this is the master transcription factor regulating autophagy and the expression of lysosome genes. When phosphorylated by mTORC1 and by ERK1/2, it cannot enter into the nucleus [49,50]. Although it could be expected that, following ERK1/2 and mTORC1 inhibition, TFEB would be less phosphorylated and more translocated to the nuclear compartment, we did not observe any significant difference in TFEB distribution following the different treatments. Then, by performing q-RT-PCR, we investigated whether autophagy genes were less transcribed and, again through this approach, we did not find significant differences among the different treatments. Thus, the slowdown of the autophagy flux was not due to a decreased transcription of autophagy genes. Then, we examined protein translation and, interestingly, we detected that Q-, Rap-, or ERK1/2-inhibition decreased this process and that combined cell treatment by U0126 + Q or U0126 + Rap further induced protein shutdown, in a manner similar to that of cycloheximide treatment. This suggests that multiple cascades are in charge for the control of protein synthesis. Indeed, ERK1/2 inhibition in Q- or Rap-treated cells not only caused a stronger inhibition of mTORC1 activity, but also increased the phosphorylation (inactivation) of eIF2α. Different stress types, such as DNA damage, misfolded protein accumulation in the ER, nutrient deprivation, and viral infection are known to cause the phosphorylation of eIF2α, resulting in the inhibition of protein translation [37,38,39]. Q, by causing the impairment of IRE1α [63], which is a main sensor of endoplasmic reticulum stress, may lead to activation of the unfolded protein response, the phosphorylation of eIF2α, and the inhibition of protein synthesis.

## 5. Conclusions

**Figure 8:**
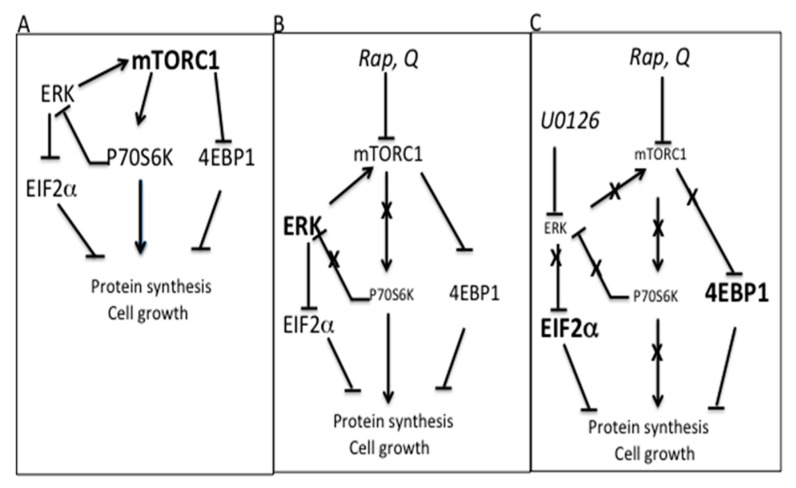
Schematic representation of the pathway leading to protein synthesis crisis. (**A**) In untreated AML cells, by phosphorylating 4EBP1, mTORC1 removes its inhibitory function on protein synthesis, while through P70S6K, mTORC1 regulates ERK activation. (**B**) Following Q or Rap removal of the inhibitory loop between mTORC1/P70S6K and ERK1/2, the MAPK causes an activation loop, resulting in mild inhibition of mTORC1 and mild de-phosphorylation of 4EBP1, with consequent partial inhibition of protein translation. Furthermore, by preventing eIF2α phosphorylation, ERK does not allow its inhibitory function on protein synthesis. (**C**) In cells treated with mTORC1 inhibitors, Rap or Q, following inhibition of ERK with U0126, the activation loop between ERK1/2 and mTORC1 is suppressed, with consequent larger inhibition of mTORC1, de-phosphorylation of 4EBP1, and inhibition of protein synthesis. ERK inhibition also leads to eIF2α phosphorylation. In this way, cells undergo a protein synthesis crisis.

Collectively, our findings indicate that in U937 and THP1 cells, there is an inhibitory loop between mTORC1/P70S6K and ERK1/2 and that ERK1/2 keeps eIF2α phosphorylation at bay (Figure 8A). Following Q or Rap treatments, the loop between mTORC1 and ERK1/2 is interrupted, ERK1/2 results are activated, the inhibition of mTORC1 is mild, and the protein translation and autophagy flux are slightly inhibited (Figure 8B). The inhibition of ERK1/2 further reduces mTORC1 activation, inducing a stressful condition, in which mTORC1 substrates are largely dephosphorylated, eIF2α is more phosphorylated, and protein synthesis is blocked (Figure 8C). By concomitantly inhibiting mTORC1 and ERK1/2, cells lose two important systems that are deputed to guarantee the amount of proteins needed to execute autophagy and prevent cell death.

Indeed, the inhibition of ERK by U0126 removes the inhibition of ERK on eIF2α and, in presence of Rap or Q, leads to suppression of the activation loop between ERK1/2 and mTORC1, allowing a stronger dephosphorylation of mTORC1 substrates (C). Due to the stronger phosphorylation of eIF2α and the dephosphorylation of 4EBP1, cells lose two important systems that are deputed to guarantee protein synthesis and cell growth. This strategy should be kept in mind when anti-AML treatments are designed. However, the role of AKT should also be considered, as this kinase is activated following the inhibition of mTORC1 and ERK1/2.

## Figures and Tables

**Table 1:** List of primers used in this study.

BECN1	for: TGGACACGAGTTTCAAGATCC
	rev: CTCCTGGGTCTCTCCTGGTT
Map1LC3b	for: GAGAAGACCTTCAAGCAGCG
	rev: AAGCTGCTTCTCACCCTTGT
SQTM1/p62	for: CCCGTCTACAGGTGAACTCC
	rev: CTGGGAGAGGGACTCAATCA
LAMP1	for: ACTACGACACCAAGAGTGGC
	rev: AAGCAATCACGAGACTGGGG
ULK1	for: TGAAAACATCGTGGCCCTGT
	rev: CCGTTGCAGTACTCCATAACC

## Data Availability

Datasets of the current study are available from the corresponding author upon reasonable request.

## Supplementary Materials

The following supporting information can be downloaded at: https://www.mdpi.com/article/10.3390/biology12050676/s1, Supplementary File: Original Images.

## References

[1] Sabatini D.M. (2017). Twenty-five years of mTOR: Uncovering the link from nutrients to growth. Proc. Natl. Acad. Sci. USA.

[2] Sabatini D.M. (2006). mTOR and cancer: Insights into a complex relationship. Nat. Rev. Cancer.

[3] Guertin D.A., Sabatini D.M. (2007). Defining the role of mTOR in cancer. Cancer Cell.

[4] Kim Y.C., Guan K.-L. (2015). mTOR: A pharmacological target for autophagy regulation. J. Clin. Invest..

[5] Oh W.J., Jacinto E. (2011). mTOR complex 2 signaling and functions. Cell Cycle.

[6] Hay N., Sonenberg N. (2004). Upstream and downstream of mTOR. Genes Dev..

[7] Mamane Y., Petroulakis E., LeBacquer O., Sonenberg N. (2006). mTOR, translation intiation and cancer. Oncogene.

[8] Pearce L.R., Komander D., Alessi D.R. (2010). The nuts and bolts of AGC protein kinases. Nat. Rev. Mol. Cell Biol..

[9] Inoki K., Li Y., Zhu T., Wu J., Guan K.L. (2002). TSC2 is phosphorylated and inhibited by Akt and suppresses mTOR signaling. Nat. Cell Biol..

[10] Manning B.D., Tee A.R., Logsdon M.N., Blenis J., Cantley L.C. (2002). Identification of the tuberous sclerosis complex-2 tumor suppressor gene product tuberin as a target of the phosphoinositide 3-kinase/Akt pathway. Mol. Cell.

[11] Garami A., Zwartkruis F.J.T., Nobukuni T., Joaquin M., Roccio M., Stocker H., Kozma S.C., Hafen E., Bos J.L., Thomas G. (2003). Insulin activation of Rheb, a mediator of mTOR/S6K/4E-BP signaling, is inhibited by TSC1 and 2. Mol. Cell.

[12] Menon S., Dibble C.C., Talbott G., Hoxhaj G., Valvezan A.J., Takahashi H., Cantley L.C., Manning B.D. (2014). Spatial control of the TSC complex integrates insulin and nutrient regulation of mTORC1 at the lysosome. Cell.

[13] Long X., Lin Y., Ortiz-Vega S., Yonezawa K., Avruch J. (2005). Rheb binds and regulates the mTOR kinase. Curr. Biol..

[14] Shaw R.J., Cantley L.C. (2006). Ras, PI(3)K and mTOR signalling controls tumour cell growth. Nature.

[15] Ma L., Chen Z., Erdjumement-Bromage H., Tempst P., Pandolfi P.P. (2005). Phosphorylation and functional inactivation of TSC2 by ERK: Implications for tuberous sclerosis and cancer pathogenesis. Cell.

[16] Ballif B.A., Roux P.P., Gerber S.A., MacKeigan J.P., Blenis J., Gygi S.P. (2005). Quantitative phosphorylation profiling of the ERK/p90 ribosomal S6 kinase-signaling cassette and its targets, the tuberous sclerosis tumor suppressor. Proc. Natl. Acad. Sci. USA.

[17] Zhang H., Bajraszewski N., Wu E., Wang H., Moseman A.P., Dabora S.L., Griffin J.D., Kwiatkowski D.J. (2007). PDGFRs are critical for PI3K/Akt activation and negatively regulated by mTOR. J.Clin. Invest..

[18] Zhang H., Cicchetti G., Onda H., Koon H.B., Asrican K., Bajraszewski N., Vazquez F., Carpenter C.L., Kwiatkowski D.J. (2003). Loss of Tsc1/Tsc2 activates mTOR and disrupts PI3K-Akt signaling through downregulation of PDGFR. J Clin. Invest..

[19] Harrington L.S., Findlay G.M., Gray A., Tolkacheva T., Wigfield S., Rebholz H., Barnett J., Leslie N.R., Cheng S., Shepherd P.R. (2004). The Tsc1-2 tumor suppressor controls insulin-PI3K signaling via regulation of IRS proteins. J. Cell Biol..

[20] Shah O.J., Wang Z., Hunter T. (2004). Inappropriate activation of the TSC/Rheb/mTOR/S6K cassette induces IRS1/2 depletion, insulin resistance, and cell survival deficiencies. Curr. Biol..

[21] O’Reilly K.E., Rojo F., She Q.B., Solit D., Mills G.B., Smith D., Lane H., Hofmann F., Hicklin D.J., Ludwig D.L. (2006). mTOR inhibition induces upstream receptor tyrosine kinase signaling and activates Akt. Cancer Res..

[22] Carracedo A., Ma L., Teruya-Feldstein J., Rojo F., Salmena L., Alimonti A., Egia A., Sasaki A.T., Thomas G., Kozma S.C. (2008). Inhibition of mTORC1 leads to MAPK pathway activation through a PI3K-dependent feedback loop in human cancer. J. Clin. Invest..

[23] Cagnol S., Chambard J.C. (2010). Erk and cell death: Mechanisms of Erk-induced cell death—Apoptosis, autophagy and senescence. FEBS J..

[24] Liu X., Ye Q., Zhao X.P., Zhang P.B., Li S., Li R.Q., Zhao X.L. (2019). RAS mutations in acute myeloid leukaemia patients: A review and meta-analysis. Clin. Chim. Acta.

[25] Boots A.W., Haenen G.R.M.M., Bast A. (2008). Health effects of quercetin: From antioxidant to nutraceutical. Eur. J. Pharmacol..

[26] Jeong J.H., An J.Y., Kwon Y.T., Rhee J.G., Lee Y.J. (2009). Effects of low dose quercetin: Cancer cell-specific inhibition of cell cycle progression. J. Cell. Biochem..

[27] Rivera Rivera A., Castilho-Pichardo L., Gerena Y., Dharmawardhane S. (2016). Anti-breast cancer potential of quercetin via the Akt/AMPK/mammalian target of rapamycin (mTOR) signaling cascade. PLoS ONE.

[28] Bruning A. (2013). Inhibition of mTOR signaling by quercetin in cancer treatment and prevention. Anticancer Agents Med. Chem..

[29] Vargas A.J., Burd R. (2010). Hormesis and synergy: Pathways and mechanisms of quercetin in cancer prevention and management. Nutrition Rev..

[30] Torello C.O., Alvarez M.C., Olalla Saad S.T. (2021). Polyphenolic flavonoid compound Quercetin effects in the treatment of acute myeloid leukemia and myelodysplastic syndromes. Molecules.

[31] Lee T.J., Kim O.H., Kim Y.H., Lim J.H., Kim S., Park J.-W., Kwon T.K. (2006). Quercetin arrests G2/M phase and induces caspase dependent cell death in U937 cells. Cancer Lett..

[32] Cheng S., Gao N., Zhang Z., Chen G., Budhraja A., Ke Z., Son Y.O., Wang X., Luo J., Shi X. (2010). Quercetin Induces Tumor-SelectiveApoptosis through Downregulation of Mcl-1 and Activation of Bax. Clin. Cancer Res..

[33] Spagnuolo C., Cerella C., Russo M., Chateauvieux S., Diederich M., Russo G.L. (2011). Quercetin downregulates Mcl-1 by acting on mRNA stability and protein degradation. Br. J. Cancer.

[34] Alvarez M.C., Maso V., Torello C.O., Ferro K.P., Saad S.T.O. (2018). The polyphenol quercetin induces cell death in leukemia by targeting epigenetic regulators of pro-apoptotic genes. Clin. Epigenet..

[35] Zhang M., Swarts S.G., Yin L., Liu C., Tian Y., Cao Y., Swarts M., Yang S., Zhang S.B., Zhang K. (2011). Antioxidant Properties of Quercetin. Adv. Exp. Med. Biol..

[36] Chen X., Dong X.S., Gao H.Y., Jing Y.F., Jin Y.L., Chang Y.Y., Chen L.Y., Wang J.H. (2016). Suppression of HSP27 increases the anti-tumor effects of quercetin in human leukemia U937 cells. Mol. Med. Rep..

[37] Proud C.G. (2019). Phosphorylation and signal transduction pathways in translational control. Cold Spring Harb. Perspect. Biol..

[38] Roux P.P., Topisirovic I. (2018). Signaling pathways involved in the regulation of mRNA translation. Mol. Cell. Biol..

[39] Wek R.C. (2018). Role of eIF2α kinases in translational control and adaptation to cellular stress. Cold Spring Harb. Perspect. Biol..

[40] Sundström C., Nilsson K. (1976). Establishment and characterization of a human histiocytic lymphoma cell line (U-937). Int. J. Cancer.

[41] Tsuchiya S., Yamabe M., Yamaguchi Y., Kobayashi Y., Konno T., Tada K. (1980). Establishment and characterization of a human acute monocytic cell line (THP-1). Int. J. Cancer.

[42] Nicoletti I., Migliorati G., Pagliacci M.C., Grignani F., Riccardi C. (1991). A rapid and simple method for measuring thymocyte apoptosis by propidium iodide staining and flow cytometry. J. Immunol. Methods.

[43] Schmidt E.K., Clavarino G., Ceppi M., Pierre P. (2009). SunSet, a nonradioactive method to monitor protein synthesis. Nat. Methods.

[44] Ghosh J., Kapur R. (2017). Role of mTORC1-S6K1 signaling pathway in regulation of hematopoietic stem cell and acute myeloid leukemia. Exp. Hematol..

[45] Pearce L.R., Alton G.R., Richter D.T., Kath J.C., Lingardo L., Chapman J., Hwang C., Alessi D.R. (2010). Characterization of PF-4708671, a novel and highly specific inhibitor of p70 ribosomal S6 kinase (S6K1). Biochem. J..

[46] Saxton R.A., Sabatini D.M. (2017). mTOR signaling in growth, metabolism and disease. Cell.

[47] Klionsky D.J., Abdelmohsen K., Abe A., Abedin M.J., Abeliovich H., Avecedo Arozena A., Adachi H., Adams C.M., Adams P.D., Adeli K. (2016). Guidelines for the use and interpretation of assays for monitoring autophagy (3th edition). Autophagy.

[48] Sardiello M., Palmieri M., Di Ronza A., Medina D.L., Valenza M., Gennarino V.A., Di Malta C., Donaudy F., Embrione V., Polishchuk R.S. (2009). A gene network regulating lysosomal biogenesis and function. Science.

[49] Settembre C., Di Malta C., Polito V.A., Arencibia M.G., Vetrini F., Erdin S., Erdin S.U., Huynh T., Medina D., Colella P. (2011). TFEB links autophagy to lysosomal biogenesis. Science.

[50] Settembre C., Zoncu R., Medina D.L., Vetrini F., Erdin S., Erdin S.U., Huynh T., Ferron M., Karsenty G., Vellard M.C. (2012). A lysosome-to-nucleus signalling mechanism senses and regulates the lysosome via mTOR and TFEB. EMBO J..

[51] Martina J.A., Chen Y., Gucek M., Puertollano R. (2012). mTORC1 functions as a transcriptional regulator of autophagy by preventing nuclear transport of TFEB. Autophagy.

[52] Roczniak-Ferguson A., Petit C.S., Froehlich F., Qian S., Ky J., Angarola B., Walther T.C., Ferguson S.M. (2012). The transcription factor TFEB links mTORC1 signaling to transcriptional control of lysosome homeostasis. Sci. Signal..

[53] Settembre C., Medina D.L. (2015). TFEB and the CLEAR network. Methods Cell Biol..

[54] Park S., Chapuis N., Tamburini J., Bardet V., Cornillet-Lefebvre P., Willems L., Green A., Mayeux P., Lacombe C., Bouscary D. (2010). Role of the PI3K/AKT and mTOR signaling pathways in acute myeloid leukemia. Haematologica.

[55] Nepstad I., Hatfield K.J., Gronningsaeter I.S., Reikvam H. (2020). The PI3K-Akt-mTOR signaling pathway in human Acute Myeloid Leukemia (AML) cells. Int. J. Mol. Sci..

[56] Carriere A., Romeo Y., Acosta-Jaquez H.A., Moreau J., Bonneil E., Thibault P., Fingar D.C., Roux P.P. (2011). ERK1/2 phosphorylate Raptor to promote Ras-dependent activation of mTOR complex 1 (mTORC1). J. Biol. Chem..

[57] Rajalingam K., Schreck R., Rapp U.R., Albert S. (2007). Ras oncogenes and their downstream targets. Biochim. Biophys. Acta.

[58] Carriere A., Cargnello M., Julien L.A., Gao H., Bonneil E., Thibault P., Roux P.P. (2008). Oncogenic MAPK signaling stimulates mTORC1 activity by promoting RSK-mediated raptor phosphorylation. Curr. Biol..

[59] Zeng X., Kinsella T.J. (2008). Mammalian target of rapamycin and S6 Kinase 1 positively regulate 6-thioguanine-induced autophagy. Cancer Res..

[60] Arico S., Petiot A., Bauvy C., Dubbelhuis P.F., Meijer A.J., Codogno P., Ogier-Denis E. (2001). The tumor suppressor PTEN positively regulates macroautophagy by inhibiting the phosphatidylinositol 3-kinase/protein kinase B pathway. J. Biol. Chem..

[61] Scott R.C., Schuldiner O., Neufeld T.P. (2004). Role and regulation of starvation-induced autophagy in the *Drosophila* fat body. Dev. Cell.

[62] Armour S.M., Baur J.A., Hsieh S.N., Land-Bracha A., Thomas S.M., Sinclair D.A. (2009). Inhibition of mammalian S6 kinase by resveratol supresses autophagy. Aging.

[63] Storniolo A., Raciti M., Cucina A., Bizzarri M., Di Renzo L. (2015). Quercetin affects Hsp70/IRE1α mediated protection from death induced by endoplasmic reticulum stress. Oxid. Med. Cell. Longev..

