# Supplementary material for: mTORC1/ERK1/2 Interplay Regulates Protein Synthesis and Survival in Acute Myeloid Leukemia Cell Lines
Source: Biology (Basel). 2023 May 2;12(5):676. doi: 10.3390/biology12050676 (PMC10215294; doi:10.3390/biology12050676)
Supplement: Supplementary file 1 [file biology-12-00676-s001.zip › biology-2266905-supplementary.pdf]

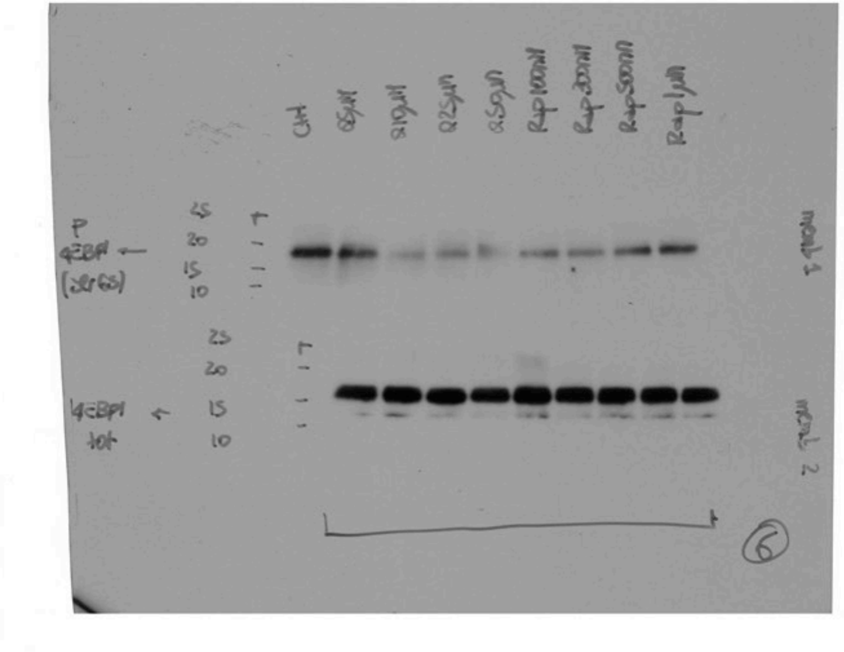

Figure 1 A
4EBP1

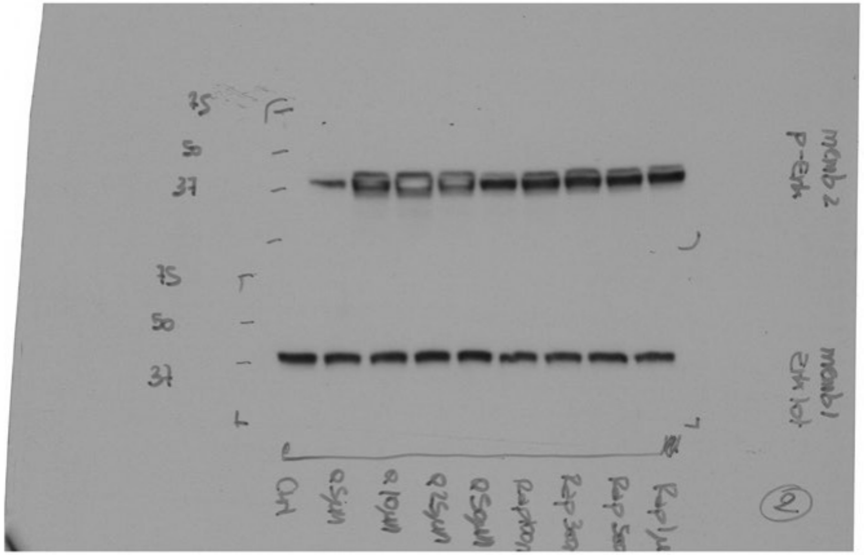

Figure 1 A
ERK

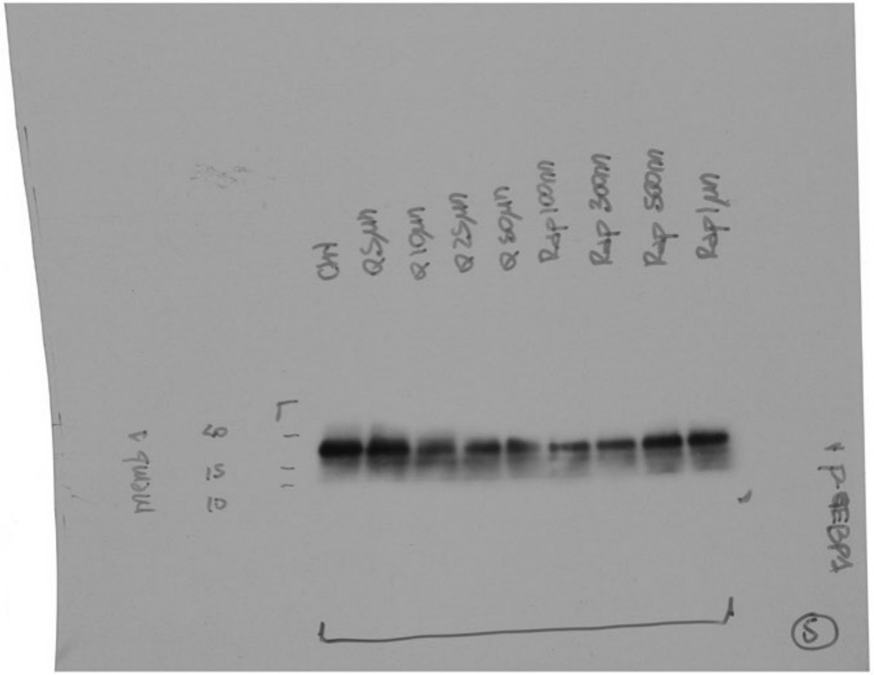

Figure 1 A
P-4EBP1

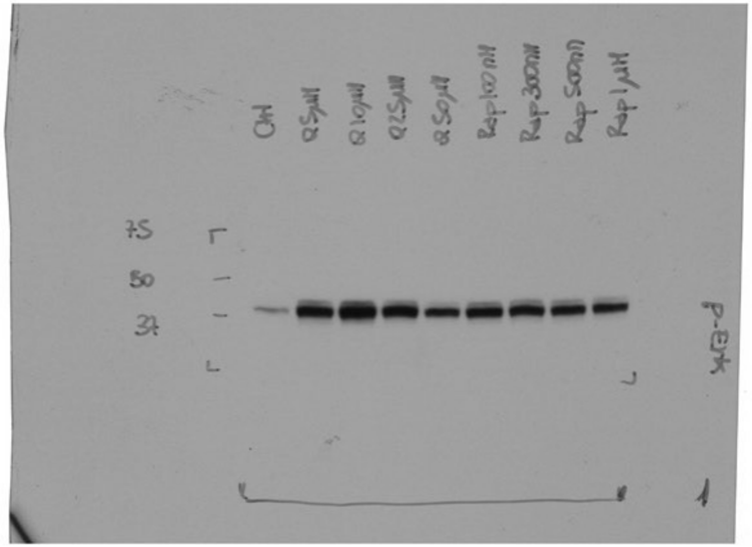

Figure 1 A
p-ERK

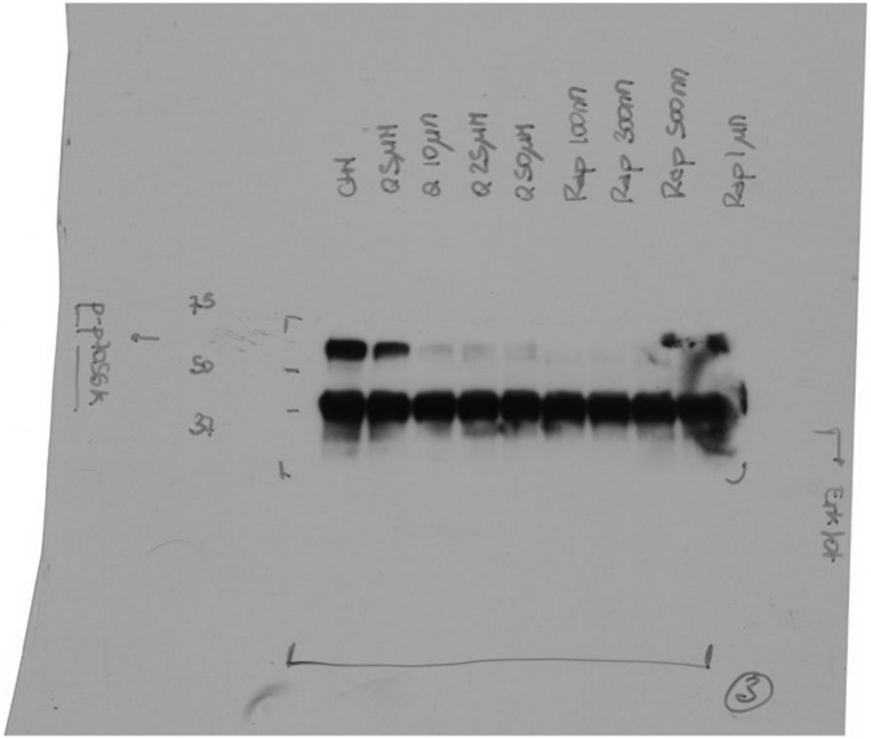

Figure 1 A
p-P70S6K

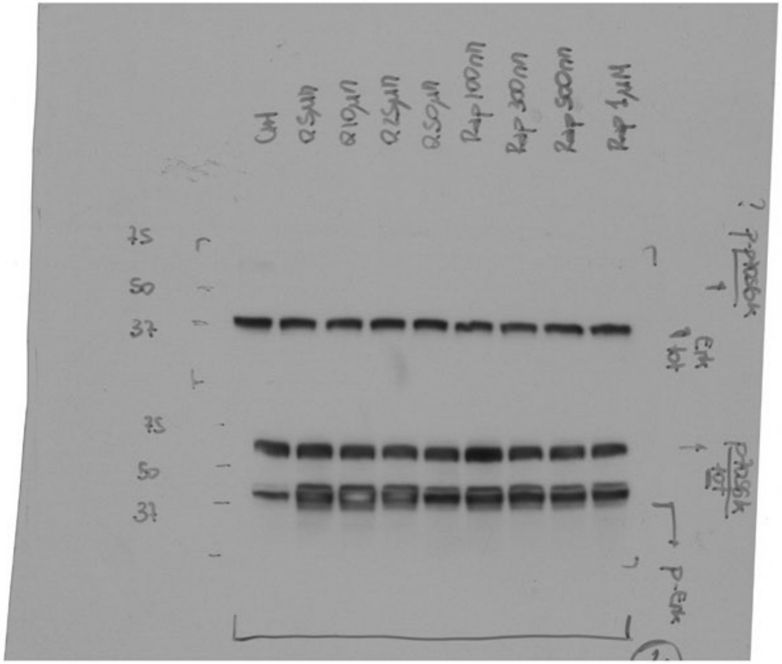

Figure 1 A
P70S6K

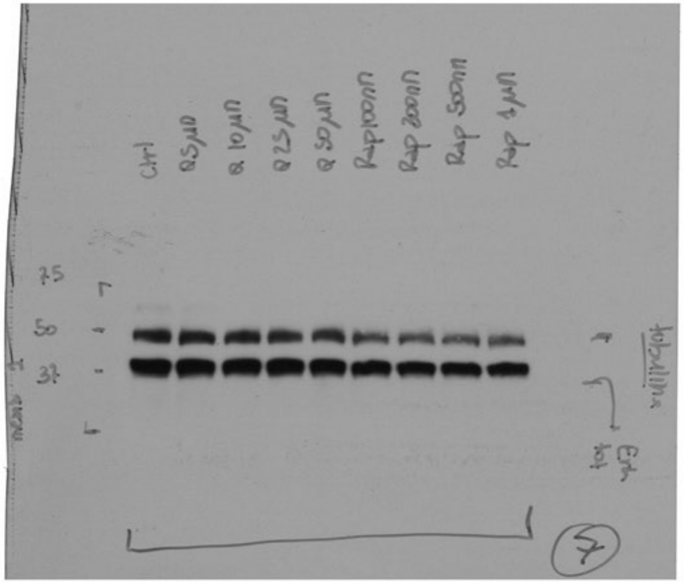

Figure 1 A
Tubulin

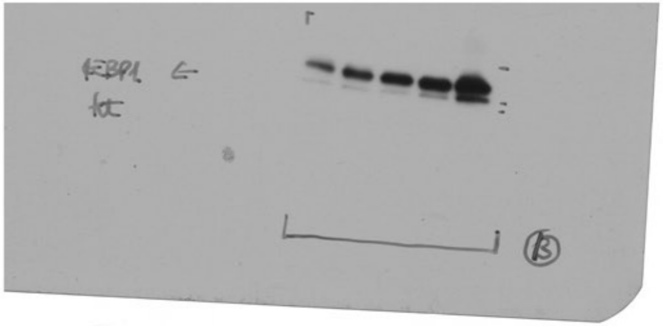

Figure 1 B
4EBP1

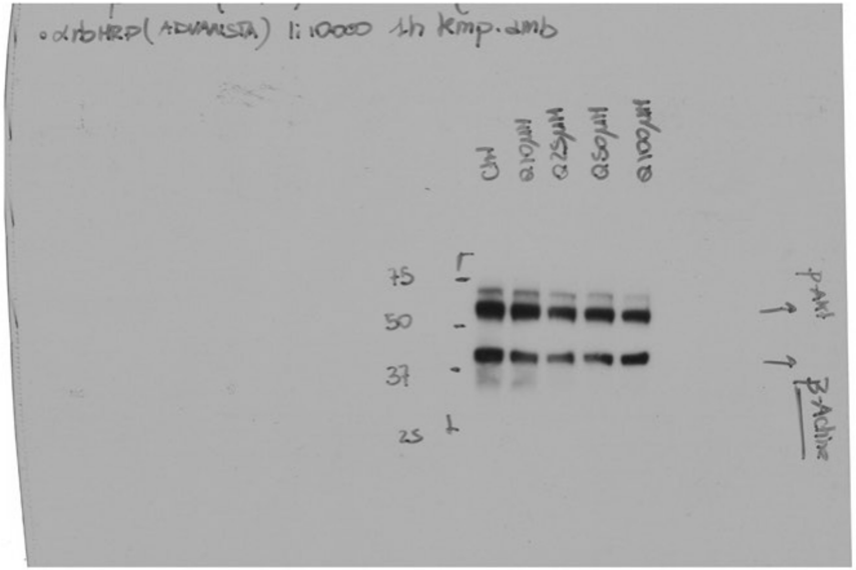

Figure 1 B
B-Actin

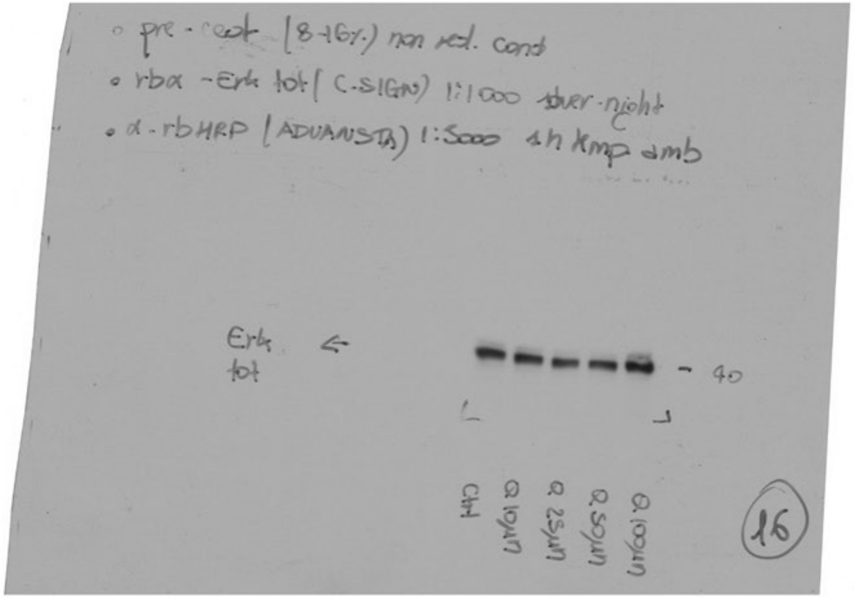

Figure 1 B
ERK

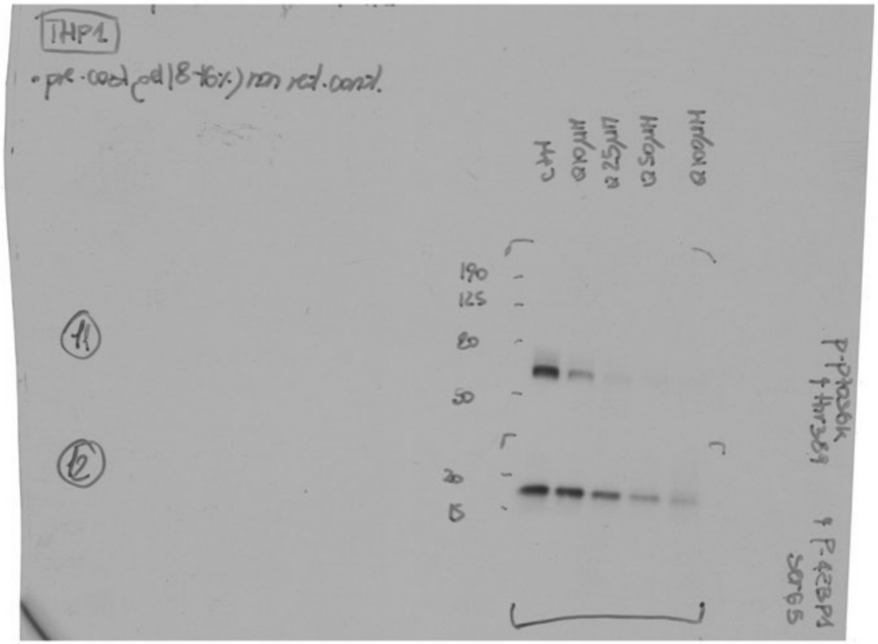

Figure 1 B
p-4EBP1

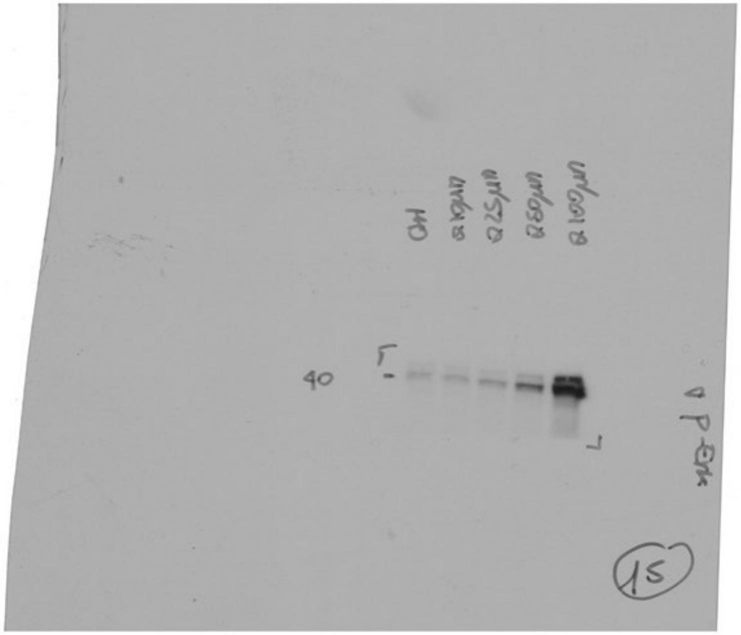

Figure 1 B
p- ERK

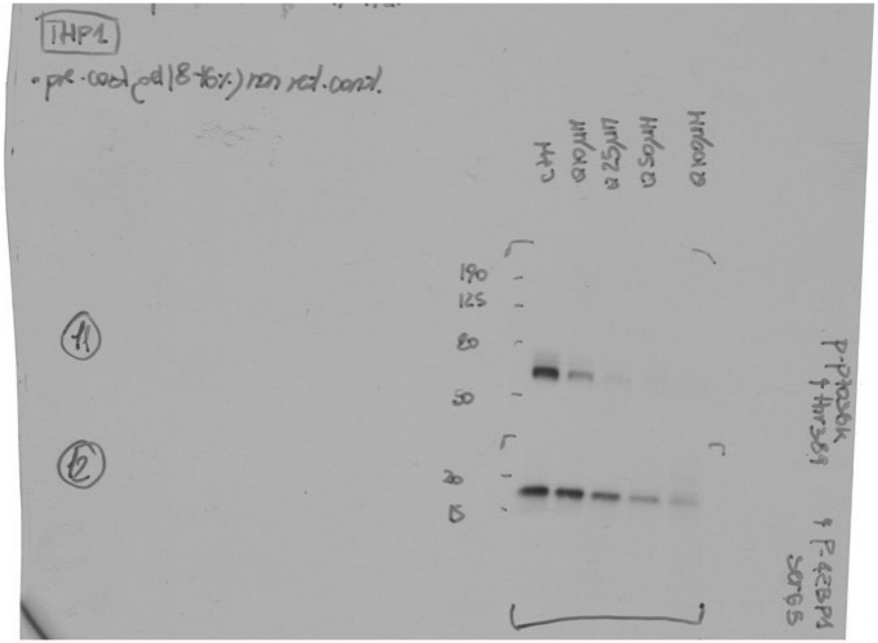

Figure 1 B
p-P70S6K

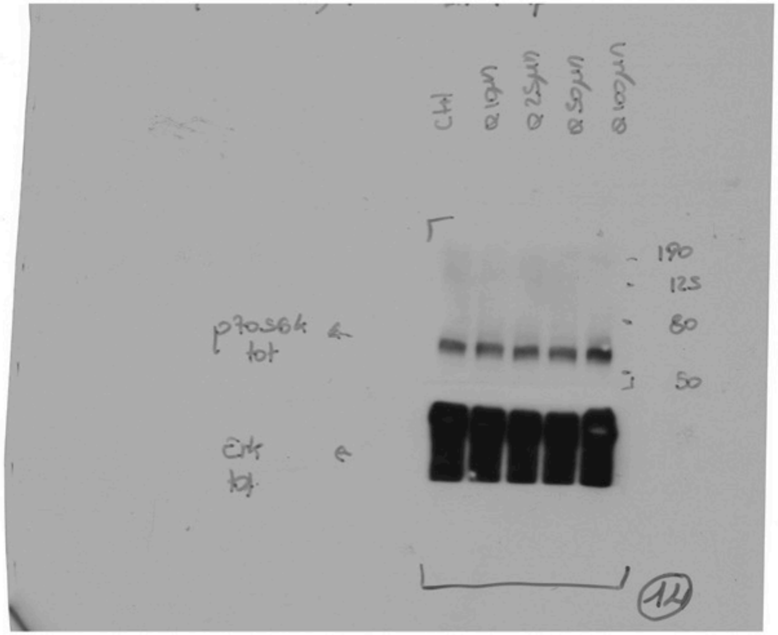

Figure 1 B
P70S6K

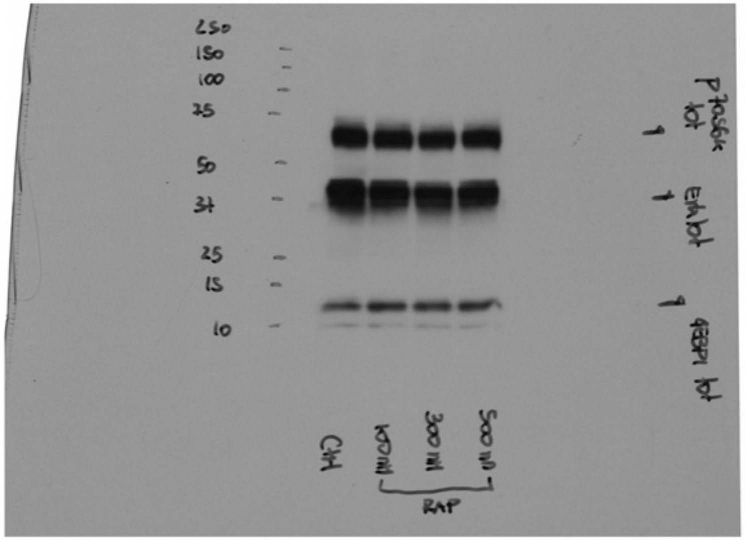

Figure 1 B
4EBP1

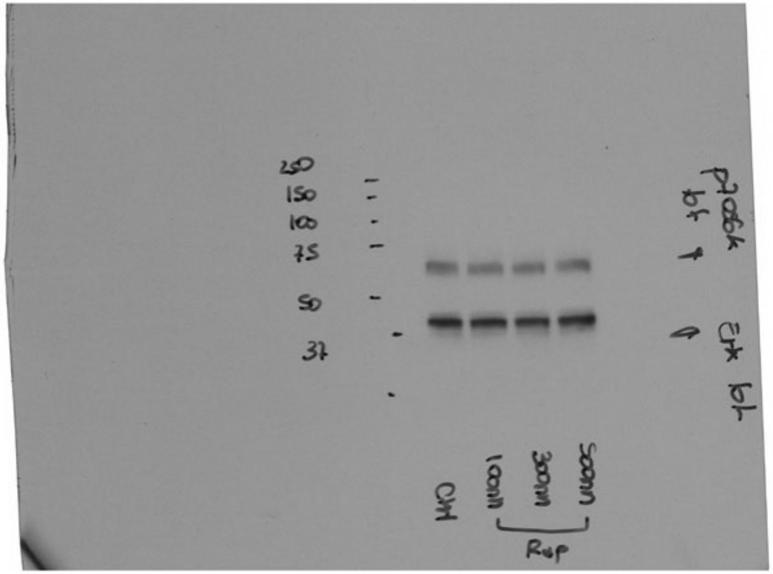

Figure 1 B
ERK

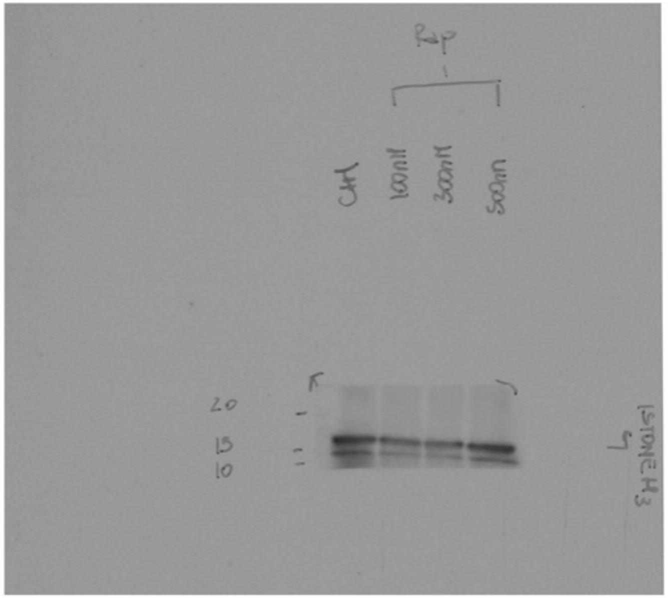

Figure 1 B
Histone H3

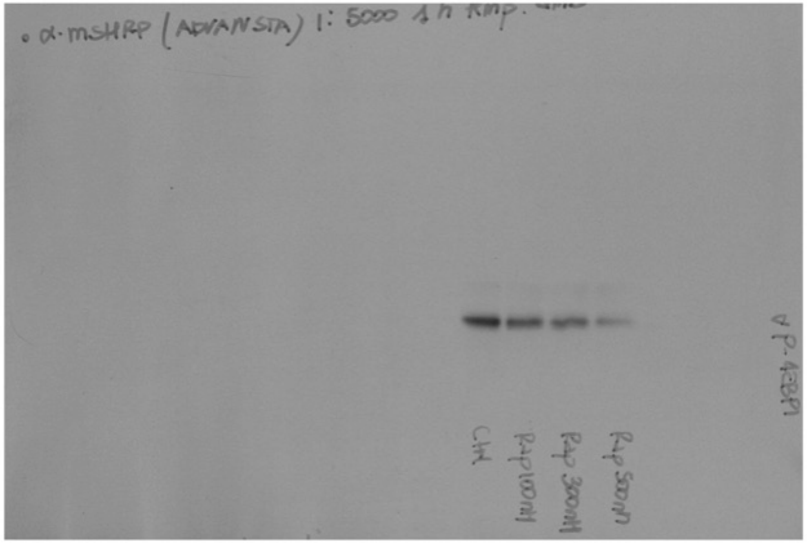

Figure 1 B
p- 4EBP1

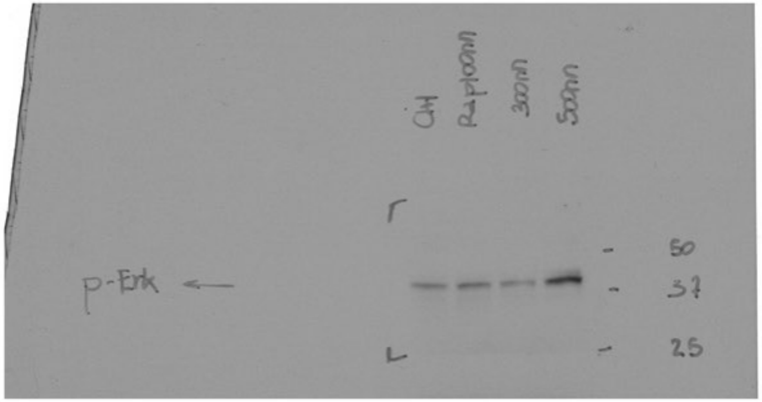

Figure 1 B
p-ERK

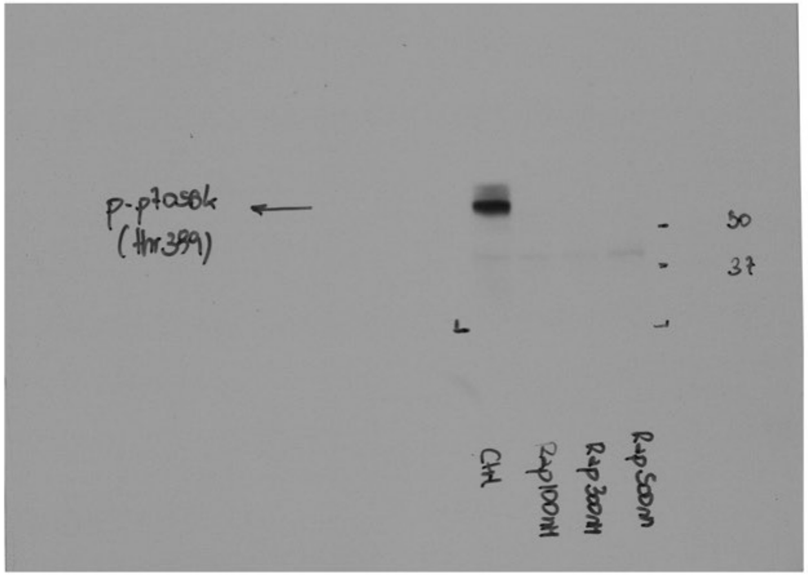

Figure 1 B
p-P70S6K

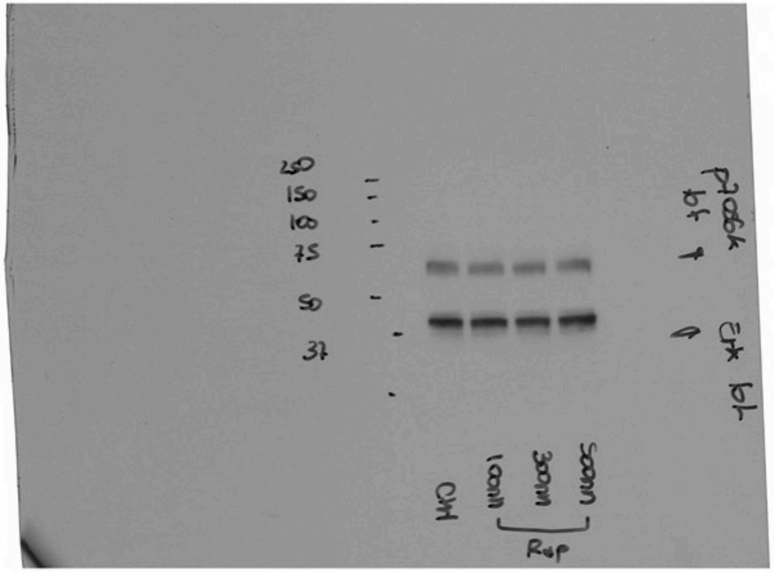

Figure 1 B
P70S6K

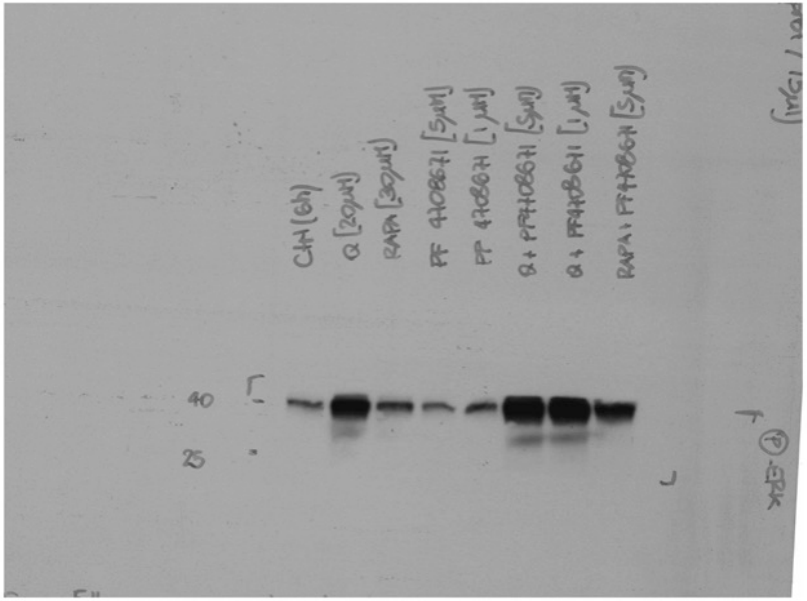

Figure 1 C
p-ERK

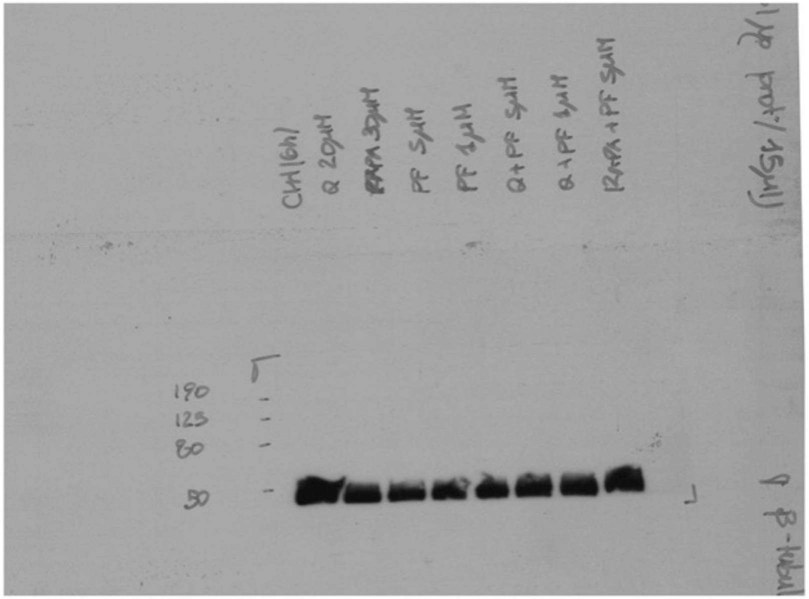

Figure 1 C
Tubulin

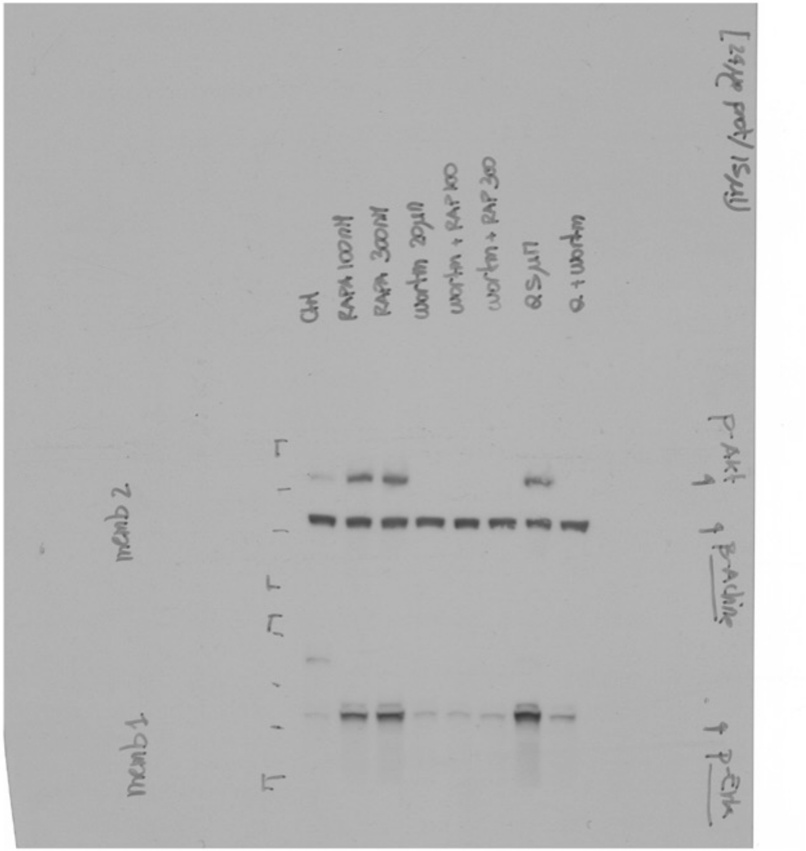

Figure 1 D
p-ERK

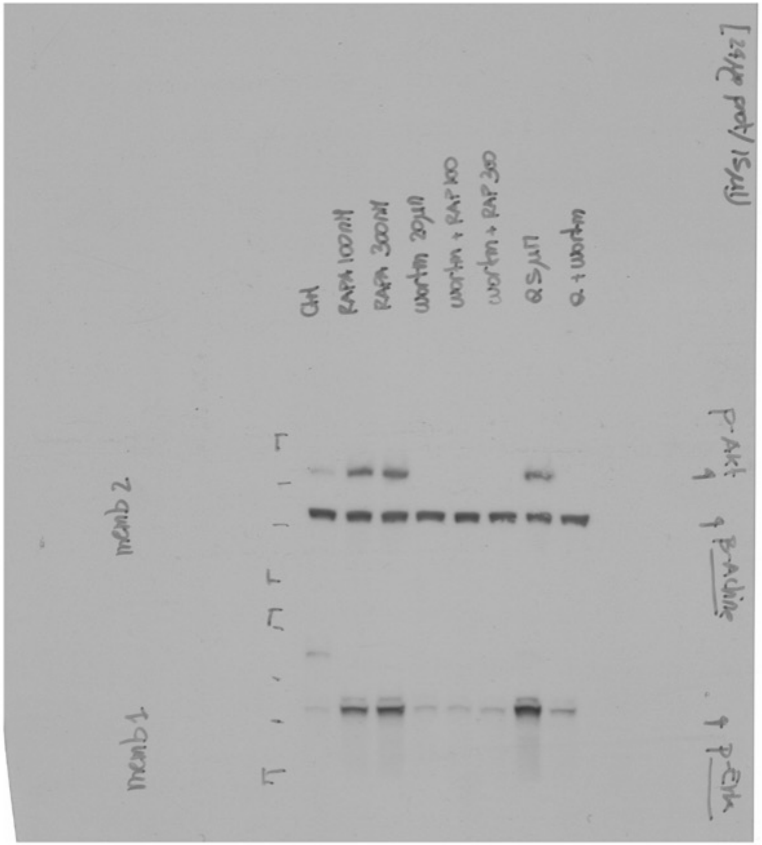

Figure 1 D
β-Actin

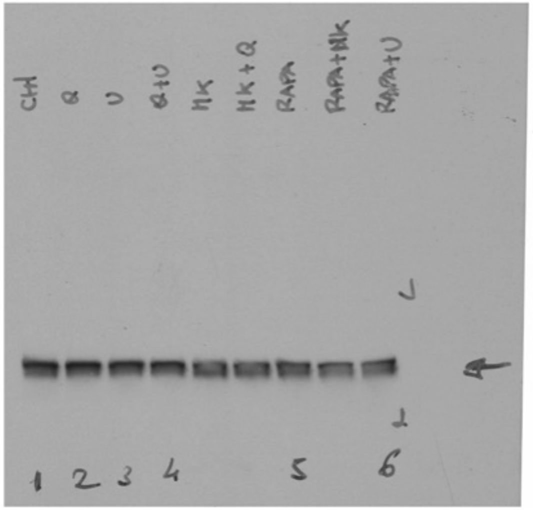

Figure 2 A
4EBP1

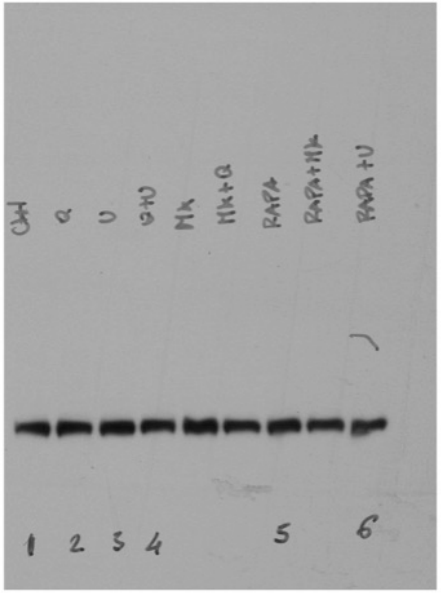

Figure 2 A
ERK

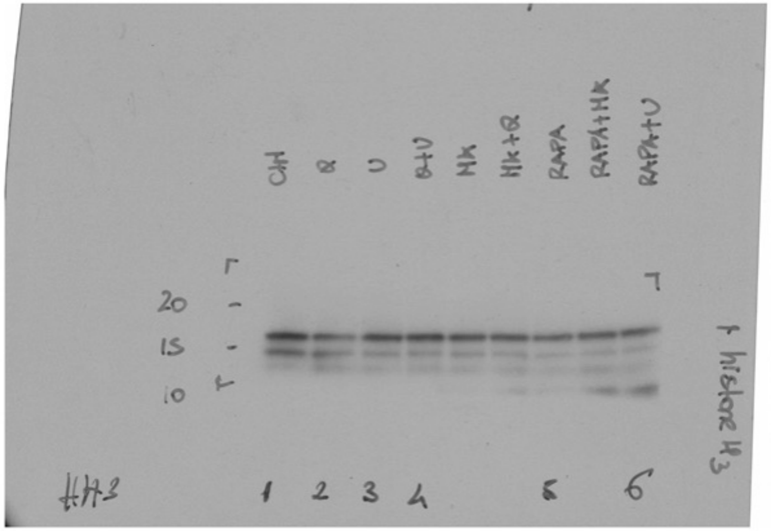

Figure 2 A
Histone H3

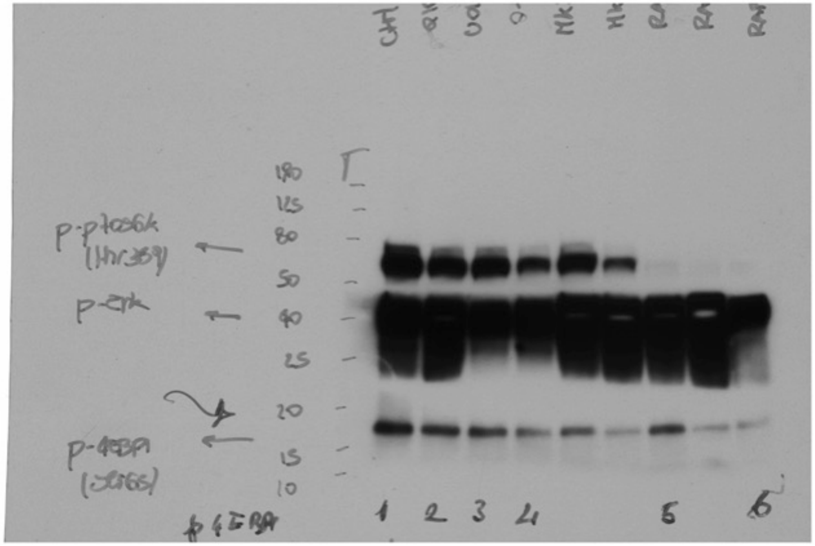

Figure 2 A
p-4EBP1

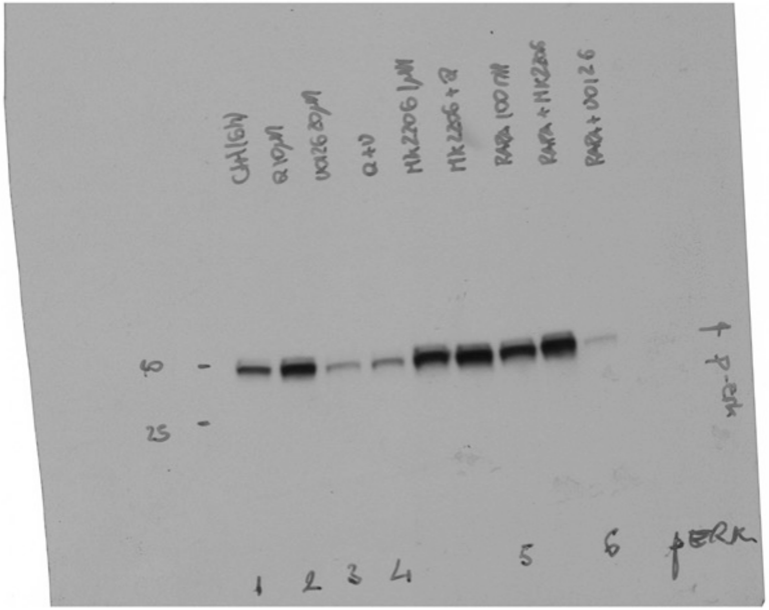

Figure 2 A
p-ERK

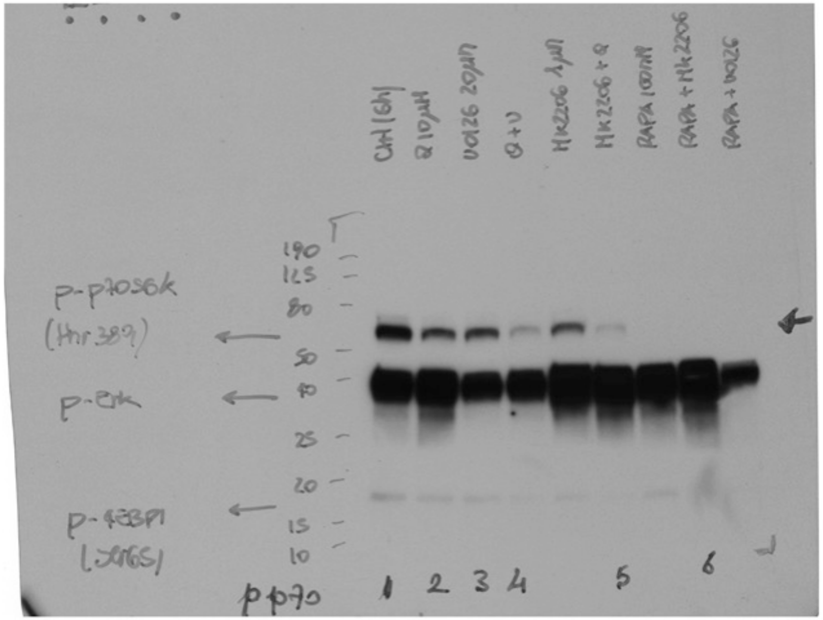

Figure 2 A
p-P70S6K

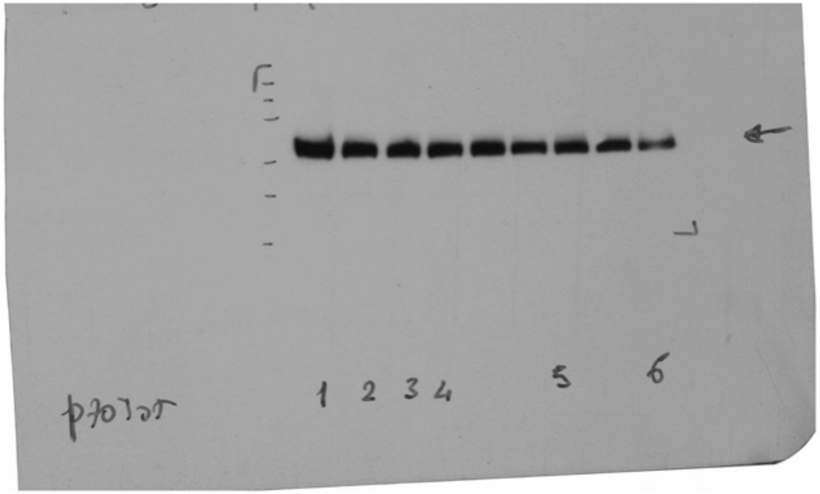

Figure 2 A
P70S6K

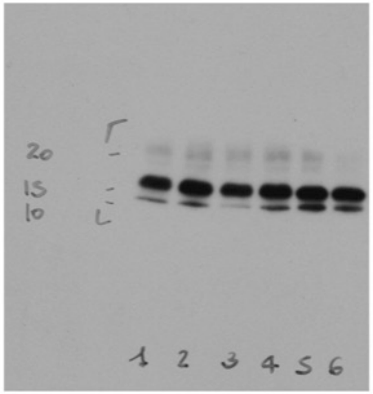

Figure 2 B
4EBP1

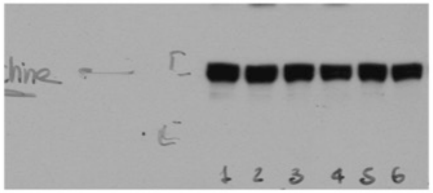

Figure 2 B
β-Actin

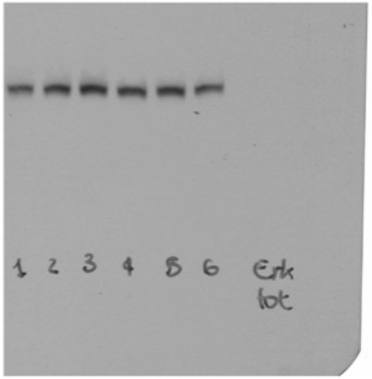

Figure 2 B
ERK

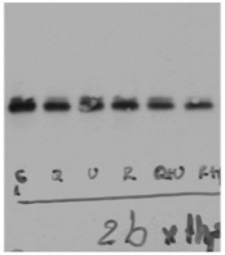

Figure 2 B
p-4EBP1

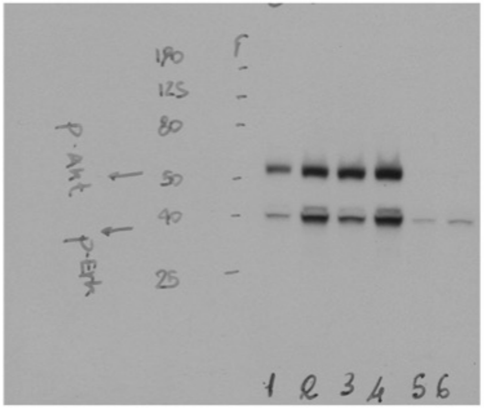

Figure 2 B
p-ERK

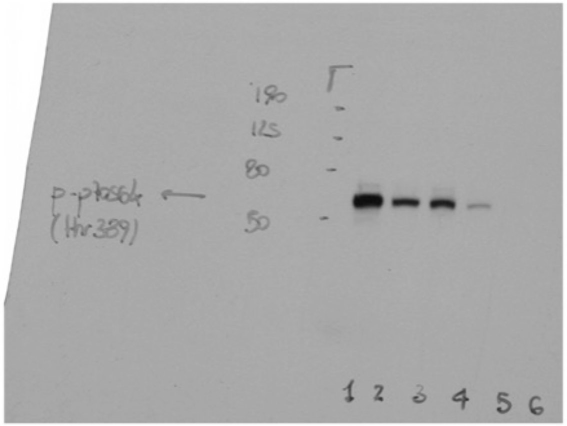

Figure 2 B
p-P70S6K

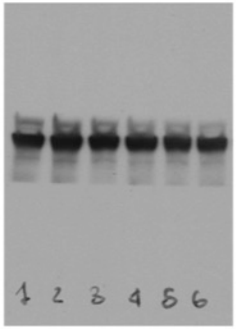

Figure 2 B
P70S6K

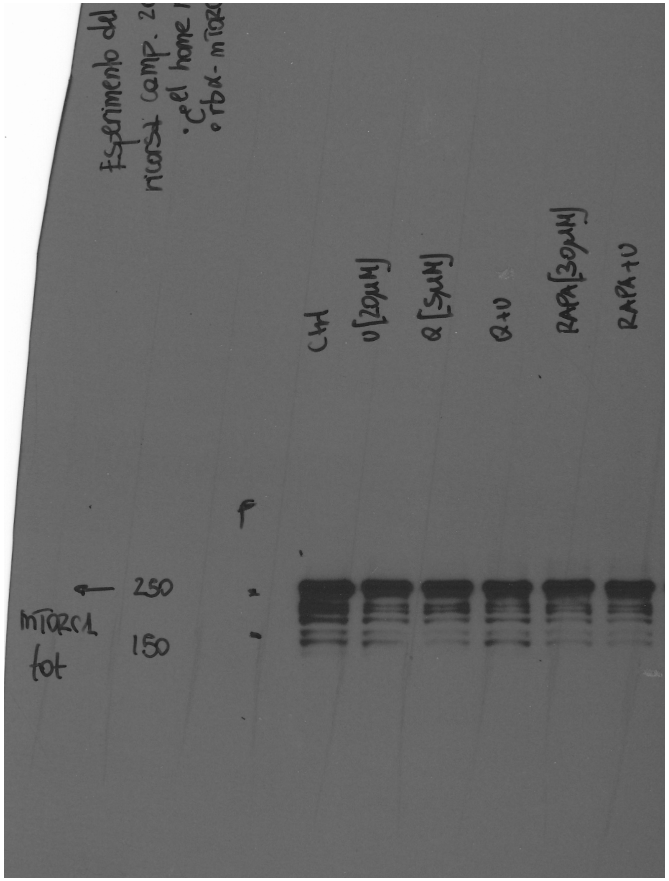

Figure 2 E
mTOR

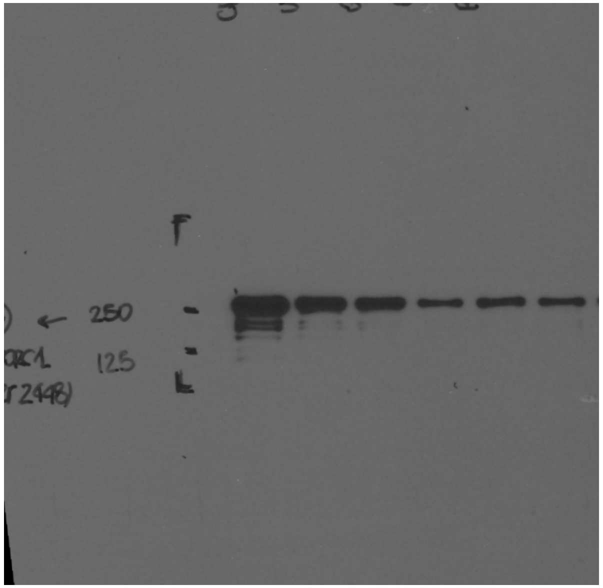

Figure 2 E
p-mTOR

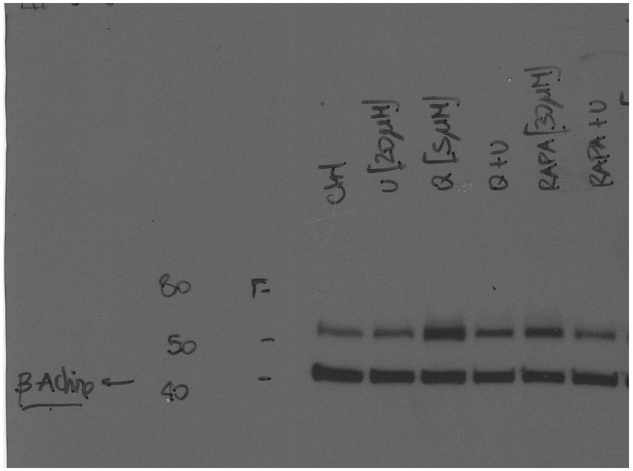

Figure 2 E
β-Actin

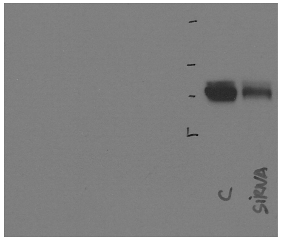

Figure 2 C
ERK

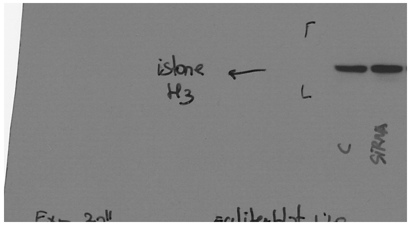

Figure 2 C
Histone H3

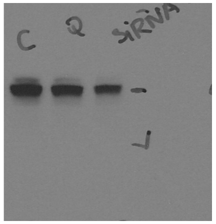

Figure 2D
P-4EBP1

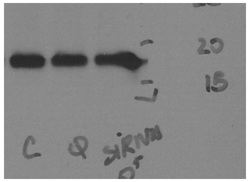

Figure 2D
4EBP1

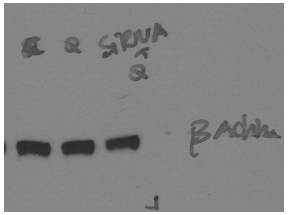

Figure 2D
β-Actin

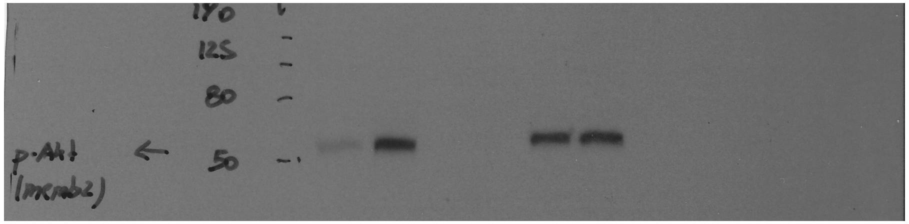

Figure 4 A
p-AKT Ser473

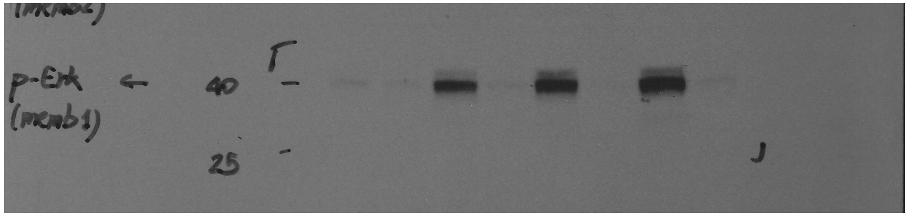

Figure 4 A
p-ERK Thr202/Tyr204

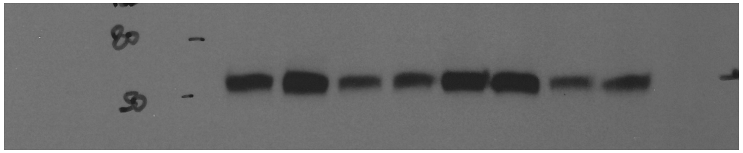

Figure 4 A
AKT

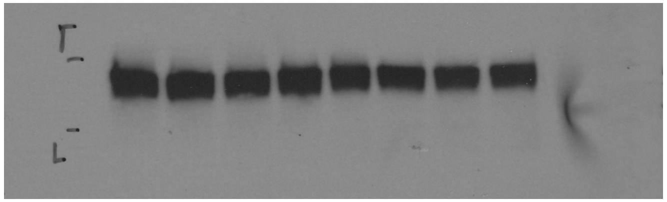

Figure 4 A
ERK

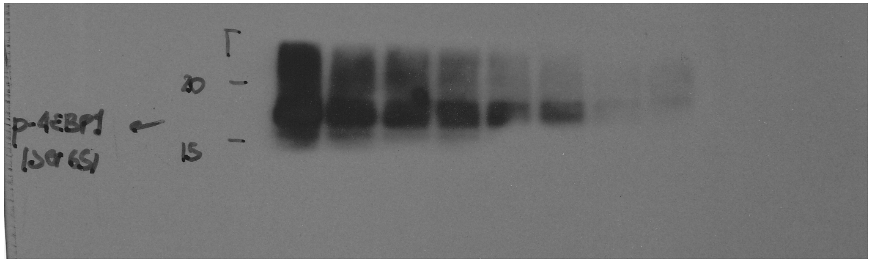

Figure 4 A
p-4EBP1 Ser65

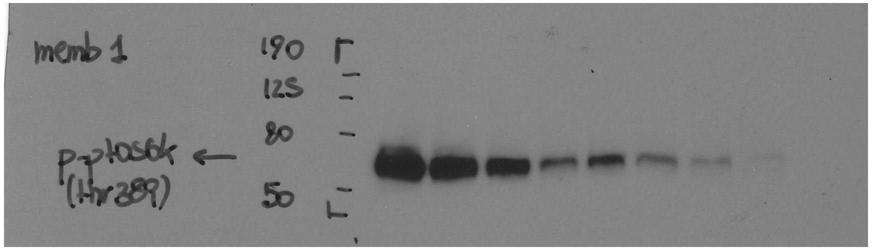

Figure 4 A
p-P70S6K Thr389

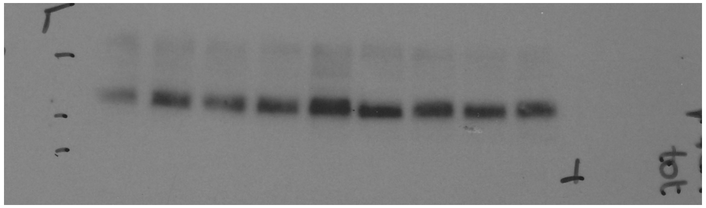

Figure 4 A
4EBP1

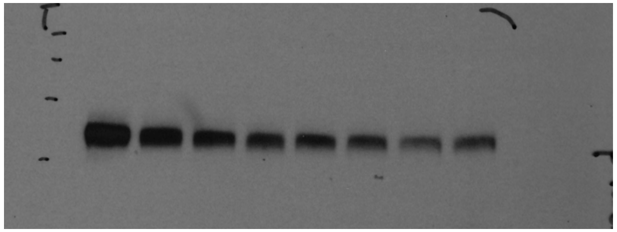

Figure 4 A
P70S6K

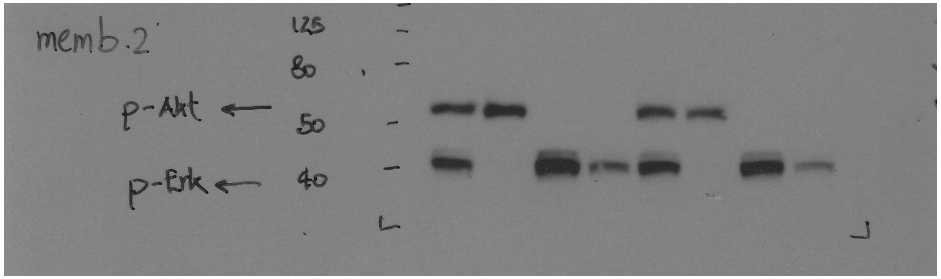

Figure 4 B
p-AKT Ser473
p-ERK Thr202/Tyr204

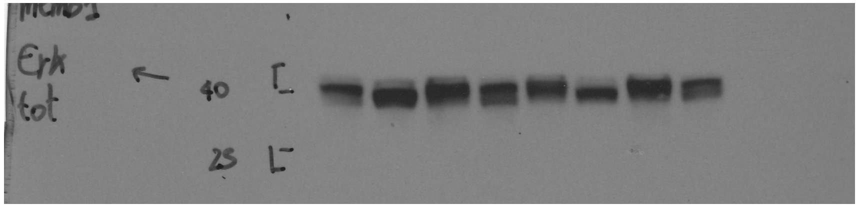

Figure 4 B
ERK

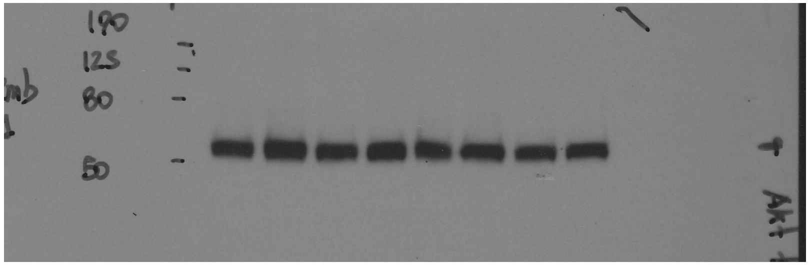

Figure 4 B
AKT

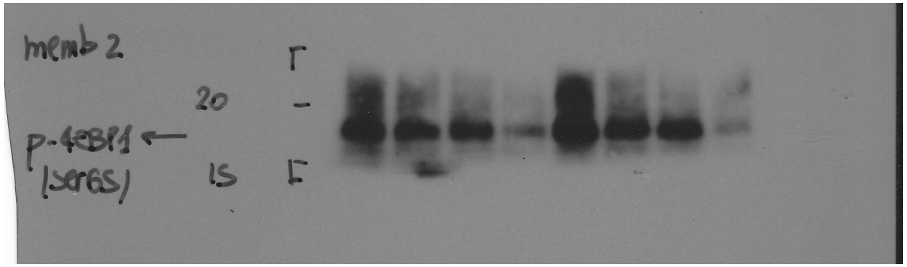

Figure 4 B
p-4EBP1 Ser65

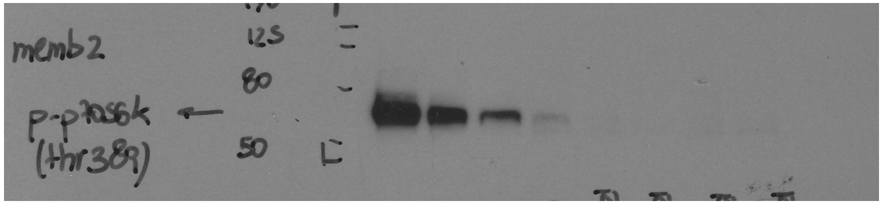

Figure 4 B
p-P70S6k Thr389

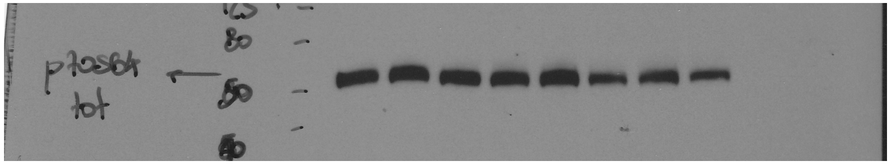

Figure 4 B
P70S6K

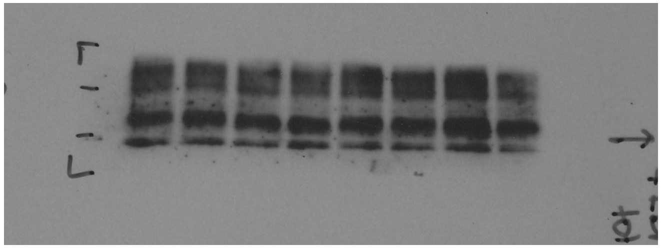

Figure 4 B
4EBP1

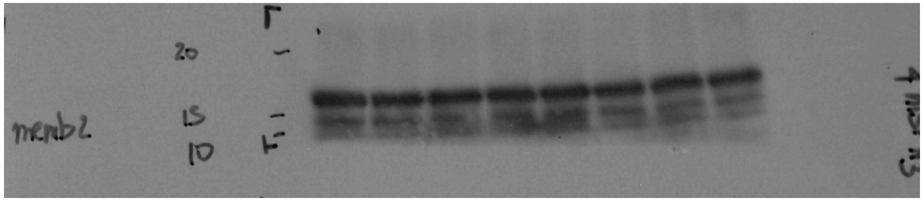

Figure 4 A
Histone H3

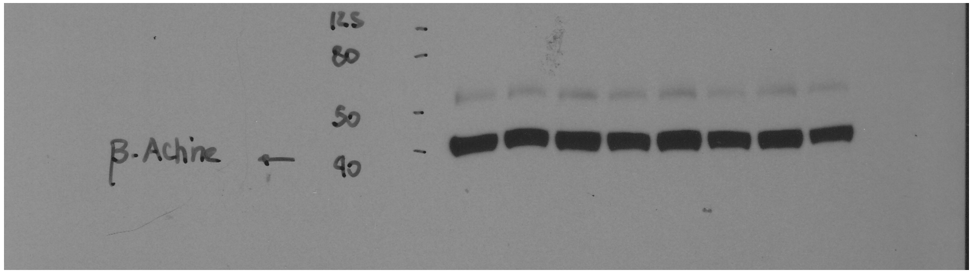

Figure 4 B
β-Actin

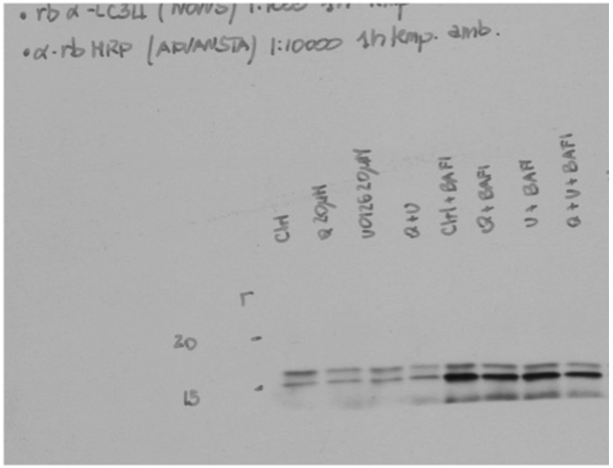

Figure 5 A
LC3 II

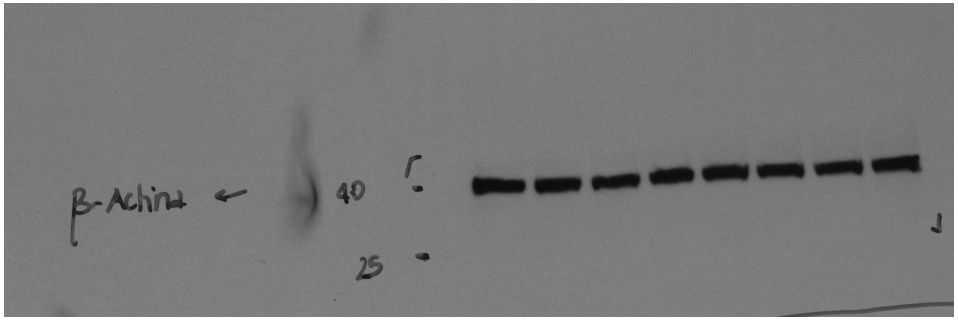

Figure 5 A
β-Actin

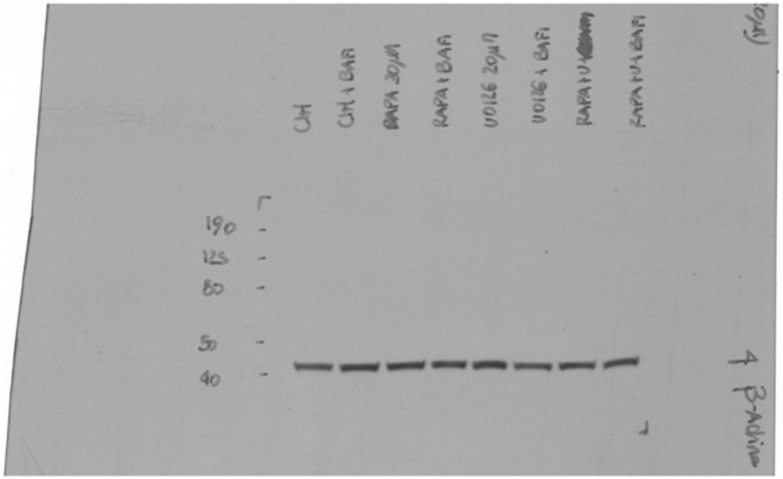

Figure 5 B
β-Actin

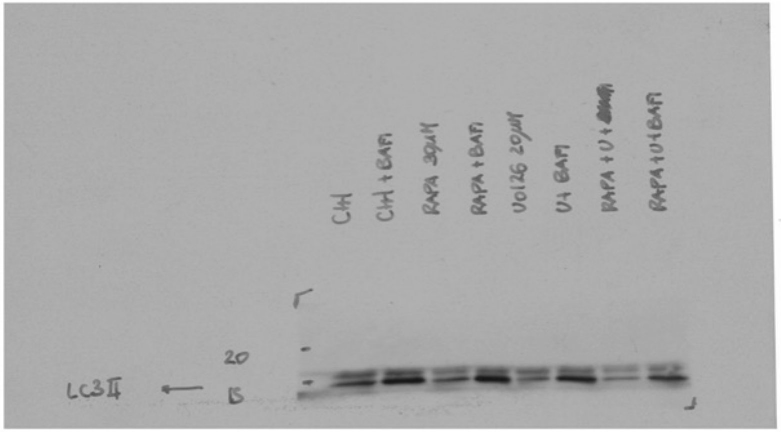

Figure 5 B
LC3 II

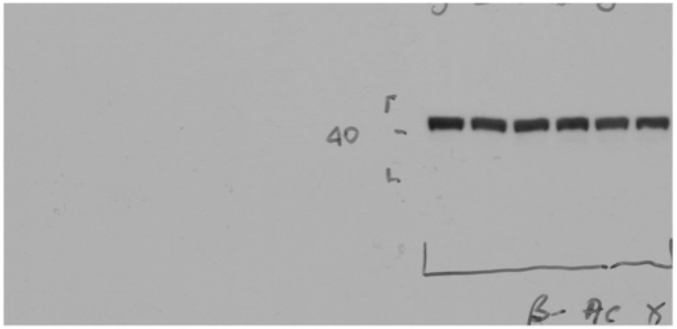

Figure 5 C
β-Actin

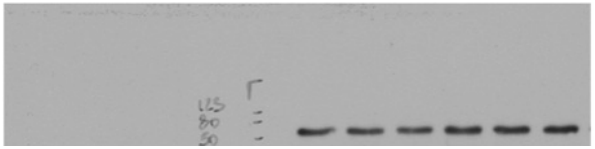

Figure 5 C
p62

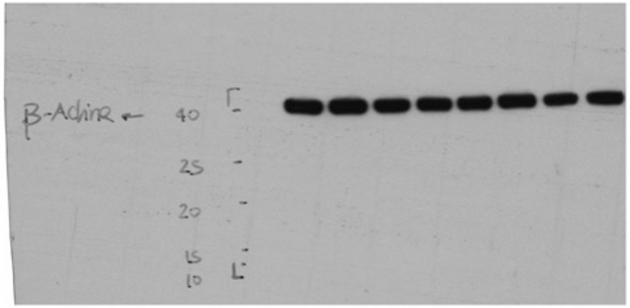

Figure 5 D
β-Actin

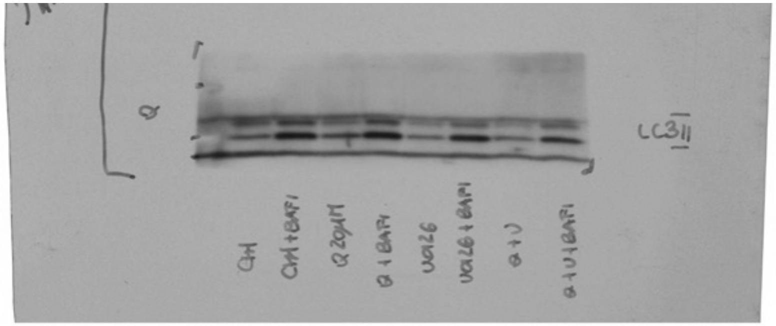

Figure 5 D
LC3 II

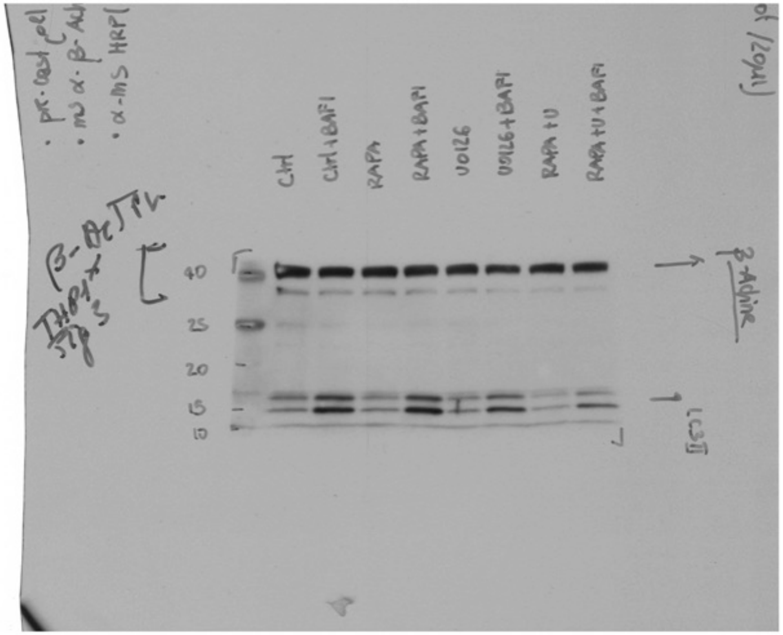

Figure 5 E
β-Actin

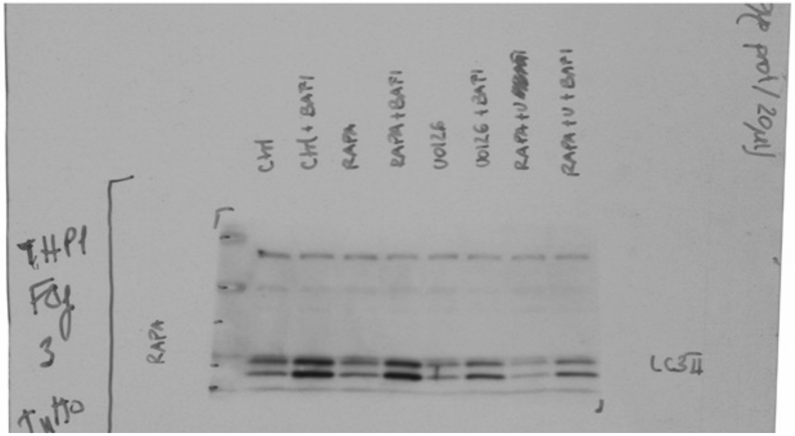

Figure 5 E
LC3 II

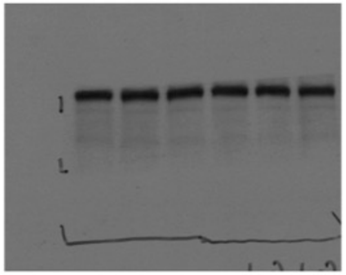

Figure 5 F
β-Actin

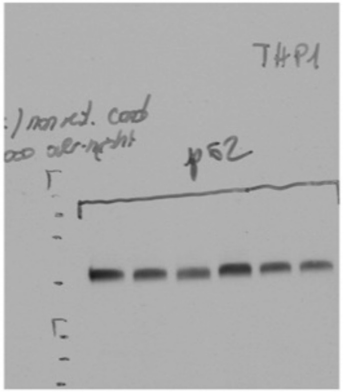

Figure 5 F
p62

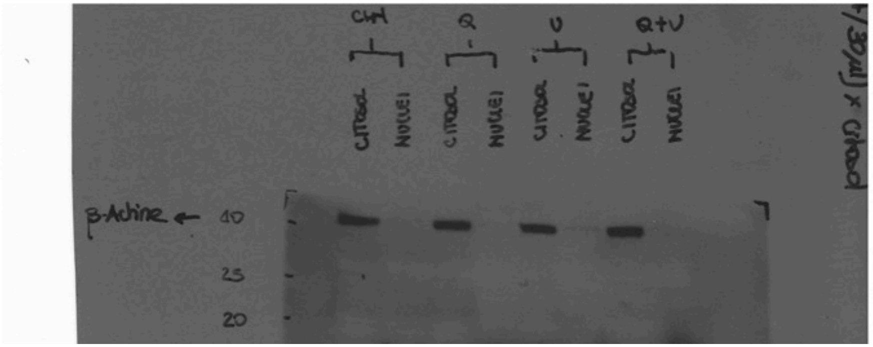

Figure 6 A
β-Actin

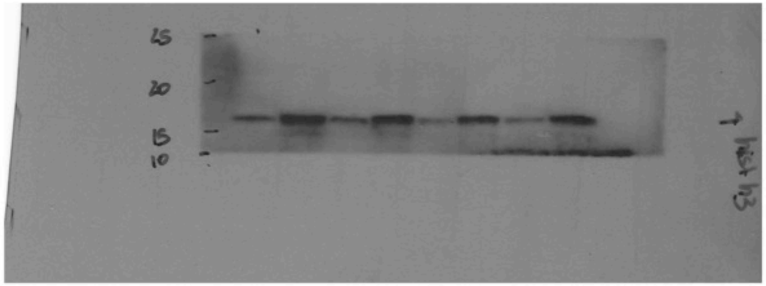

Figure 6 A
Histone H3

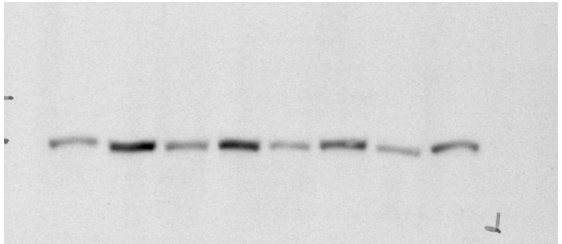

Figure 6 A
Lamin B

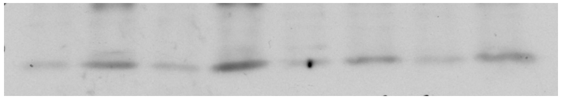

Figure 6 B
Lamin B

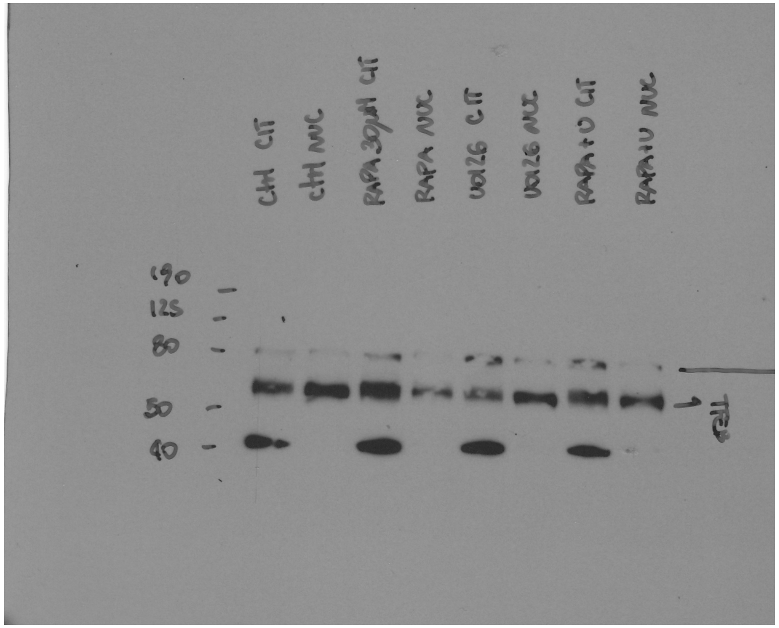

Figure 6 B
TFEB, β-Actin

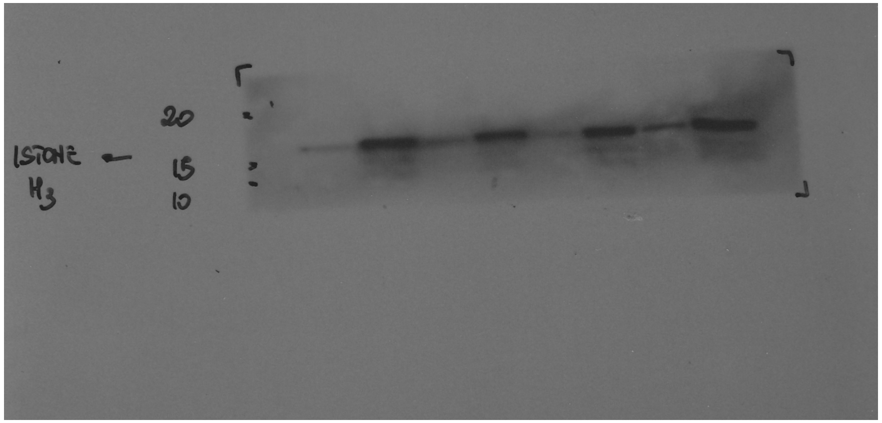

Figure 6 B
Histone H3

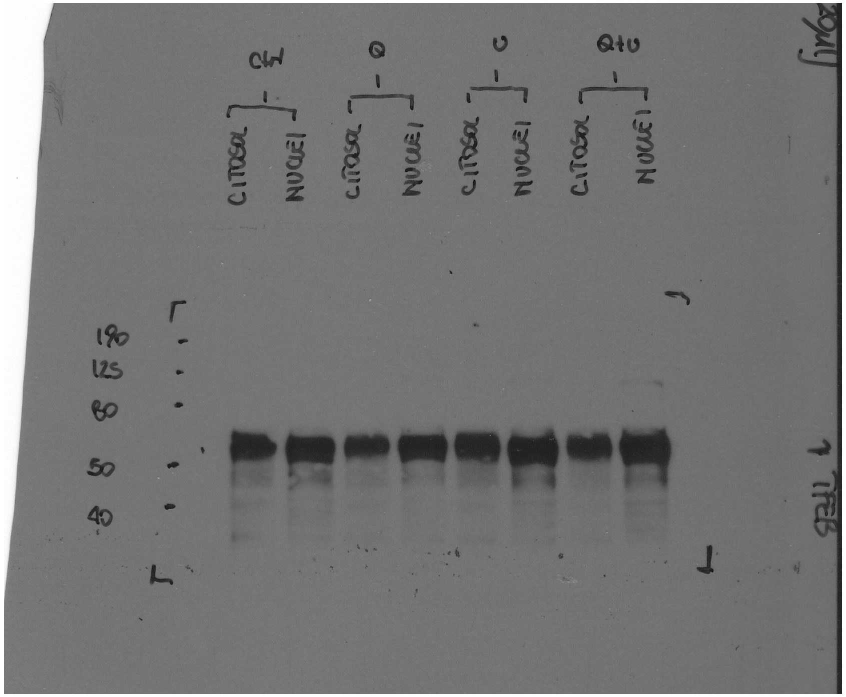

Figure 6 A
TFEB

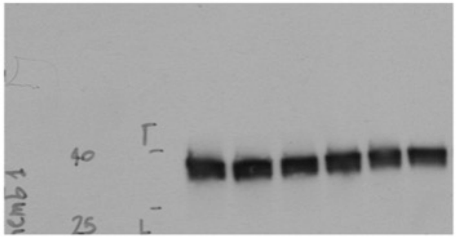

Figure 7 A
eIF2α

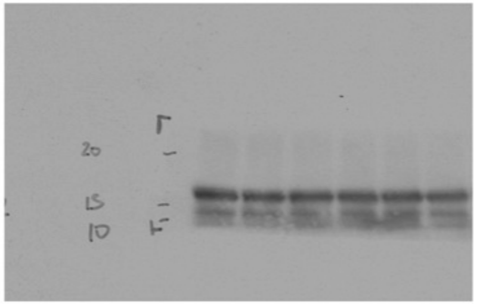

Figure 7 A
Histone H3

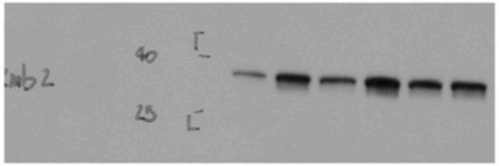

Figure 7 A
p-eIF2α

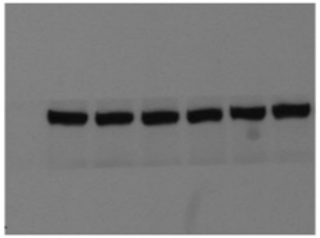

Figure 7 B
eIF2α

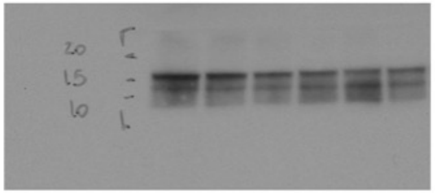

Figure 7 B
Histone H3

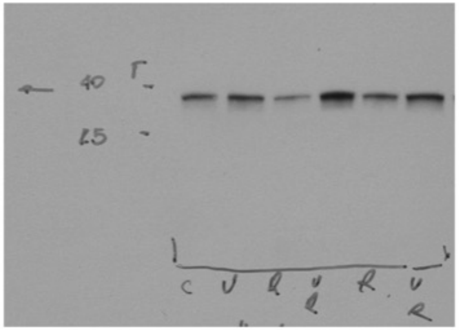

Figure 7 B
p-eIF2α

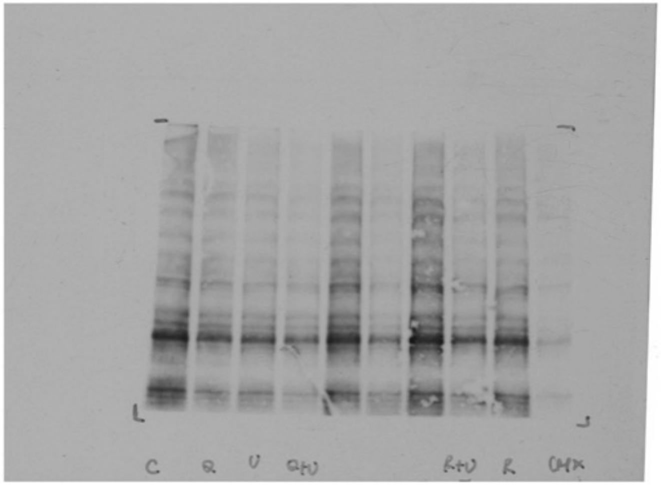

Figure 7 C

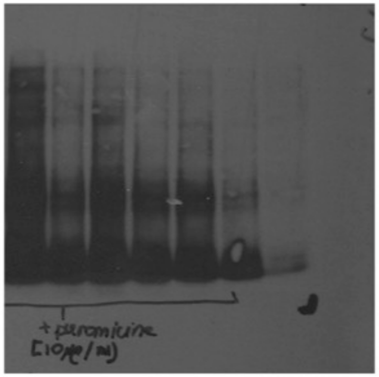

Figure 7 D
